# High-dose intravenous vitamin C, a promising multi-targeting agent in the treatment of cancer

**DOI:** 10.1186/s13046-021-02134-y

**Published:** 2021-10-30

**Authors:** Franziska Böttger, Andrea Vallés-Martí, Loraine Cahn, Connie R. Jimenez

**Affiliations:** Department of Medical Oncology, Cancer Center Amsterdam, OncoProteomics Laboratory, Amsterdam UMC, Location VU University Medical Center, 1081 HV Amsterdam, the Netherlands

**Keywords:** Ascorbic acid, Vitamin C, IVC, Cancer, Clinical trials, Proteomics, Transcriptomics, Metabolomics

## Abstract

Mounting evidence indicates that vitamin C has the potential to be a potent anti-cancer agent when administered intravenously and in high doses (high-dose IVC). Early phase clinical trials have confirmed safety and indicated efficacy of IVC in eradicating tumour cells of various cancer types. In recent years, the multi-targeting effects of vitamin C were unravelled, demonstrating a role as cancer-specific, pro-oxidative cytotoxic agent, anti-cancer epigenetic regulator and immune modulator, reversing epithelial-to-mesenchymal transition, inhibiting hypoxia and oncogenic kinase signalling and boosting immune response. Moreover, high-dose IVC is powerful as an adjuvant treatment for cancer, acting synergistically with many standard (chemo-) therapies, as well as a method for mitigating the toxic side-effects of chemotherapy. Despite the rationale and ample evidence, strong clinical data and phase III studies are lacking. Therefore, there is a need for more extensive awareness of the use of this highly promising, non-toxic cancer treatment in the clinical setting. In this review, we provide an elaborate overview of pre-clinical and clinical studies using high-dose IVC as anti-cancer agent, as well as a detailed evaluation of the main known molecular mechanisms involved. A special focus is put on global molecular profiling studies in this respect. In addition, an outlook on future implications of high-dose vitamin C in cancer treatment is presented and recommendations for further research are discussed.

## Background

Vitamin C (VitC), also known as ascorbic acid or ascorbate, is an essential water-soluble vitamin that plays an important role in human physiology. Most of its physiological functions involve its ability to act as an antioxidant or as a cofactor for a wide variety of enzymatic reactions, thereby contributing to stabilisation of the tertiary structure of collagen, norepinephrine synthesis and iron absorption [1, 2]. Emerging data show that VitC is also a cofactor for newly characterised hydroxylases of the family of Fe-containing 2-oxoglutarate-dependent dioxygenases that regulate gene transcription and cell signalling pathways [3, 4]. In addition, immune cells accumulate high concentrations of VitC, underlining its key function in various processes within the immune system [5]. Importantly, while most vertebrate species can synthesize ascorbic acid, humans cannot, and they are therefore dependent on oral consumption of VitC.

The concept of utilizing VitC as a therapeutic agent for cancer care was first introduced by double Nobel Prize winning chemist Linus Pauling and physician Ewan Cameron almost 50 years ago [6–8]. Specifically, Pauling and Cameron published a number of clinical reports that indicated significantly prolonged survival rates of terminal cancer patients treated with pharmacological doses of VitC (10 g/ day by intravenous infusion for about 10 days and orally thereafter) compared to matched historical controls that did not receive VitC. The same amounts of high-dose VitC administered orally only in randomized double blind placebo control studies could not confirm this favourable response in advanced human cancer [9, 10]. Herein lies the essence of much controversy concerning the implementation of VitC in cancer treatment over the past decades. An important distinction must therefore be made between orally administered VitC (OC), achieving maximum plasma concentrations of no more than 220 μmol/L of blood, and pharmacological or high-dose IVC, generating plasma concentrations up into the millimolar range (≥ 15 mmol/L) [11–13], which is needed to kill cancer cells based on pre-clinical studies.

In light of this, high-dose IVC has re-emerged as a potent anti-cancer agent over the past two decades, with several phase I and a few phase II clinical trials reporting high tolerability and safety with promising signs of efficacy in the treatment of various cancer types, either as monotherapy or as a combination therapy [14–16]. In addition, there is strong clinical evidence for IVC’s ability to reduce chemotherapy-related side effects, such as fatigue, and to improve quality of life also in the palliative care setting [17–19].

The aim of this review is to create an up-to-date overview of the most important research conducted within the field of high-dose VitC and cancer therapy. First, the use of high-dose VitC mono- and combination therapy in the pre-clinical and clinical setting is discussed, followed by a discussion of the molecular mechanisms found to be involved in the anti-cancer activity portrayed by VitC. Specifically, the contribution of emerging global profiling studies based on proteomics, transcriptomics and metabolomics to these insights will be highlighted. In this regard, our findings will provide an outlook on future research, examining current gaps in our knowledge and addressing the limitations of research in the clinical setting and the need for more extensive clinical trials. Also, future implications of high-dose VitC in cancer therapy will be discussed in both treatment and palliative care.

## High-dose VitC as a single agent

The pioneering clinical studies that initiated the interest in VitC as anticancer agent [6–8] employed VitC as single agent. Since then, a great number of clinical and pre-clinical studies have explored high-dose VitC. In this section, we briefly summarize the pre-clinical and clinical studies of VitC as monotherapy before elaborating more on the combination therapy studies.

### Pre-clinical VitC monotherapy studies

A vast number of studies have shown encouraging anti-cancer activity of VitC at millimolar concentrations (~ 1–20 mM) in pre-clinical models of various cancer types [15]. The most investigated have been leukaemia [20–24], colon cancer [25–32], melanoma [33–37], pancreatic cancer [14, 31, 38] and prostate cancer [39–41]. Similar results have been described for the treatment of non-small-cell lung cancer (NSCLC) [16], breast cancer [31, 42], ovarian cancer [31, 43, 44], hepatocellular carcinoma [45, 46], malignant mesothelioma [47, 48], thyroid cancer [49, 50], oral squamous cell carcinoma [51], neuroblastoma [52] and glioma, including the difficult-to-treat glioblastoma multiform (GBM) [16, 53, 54].

One notable example of the progress in VitC pre-clinical research is the recent work in hard-to-treat Kirsten Rat Sarcoma Viral Oncogene Homolog (KRAS) driven tumours, such as KRAS mutant colorectal cancer (CRC) [25, 27, 32]. Based on prior studies by Yun et al. [32] and Aguilera et al. [25], Cenigaonandia-Campillo et al. [27] used elevated doses of VitC (5–10 mM) in KRAS mutant CRC tumours, both in vitro and in vivo. They showed that VitC was able to target common metabolic aberrancies by decreasing adenosine triphosphate (ATP) and glucose transporter 1 (GLUT-1) levels, as well as by dissipating the mitochondrial membrane potential, which could sensitize KRAS mutant CRC cells to current treatments such as chemotherapy. Given the importance of developing better treatments for patients with KRAS driven tumours, non-toxic combinations with VitC are also being explored and will be discussed in the following section 2.

In the majority of cancer types, most of the in vivo studies have shown inhibition of tumour growth (40–60%) by using elevated doses of ascorbate (1-4 g/kg) either intravenously (IV) or intraperitoneally (IP) [15, 55–57]. Importantly, in order to maintain VitC levels inside the tumour, daily administration is the most optimal schedule [56]. By using these doses and frequency, VitC also successfully reduced and/or impaired metastasis formation (50–90%) [33, 39, 43, 58–61].

In terms of safety and tolerability, several studies have shown that high-dose VitC does not increase toxicity levels in vivo yet protects from other treatment side-effects when used as an adjuvant agent [15, 62–64].

Overall, the studies performed in vitro and in vivo using high-dose VitC as single agent in a large number of cancer types, have shown that it is a promising anti-cancer agent impairing both tumor growth and metastasis.

### Clinical VitC monotherapy studies

Clinical monotherapy studies administering high-dose VitC in patients with various types of advanced malignancies report this therapy to be safe, showing no significant toxicity at doses of up to 3 g/kg [13] (Table 2). These studies additionally demonstrated that at the given doses, ascorbate plasma levels of over 10 mM could be sustained for several hours, and observed maximum achievable blood concentrations of up to 49 mM [13]. Grade 3 or higher adverse events possibly related to IVC treatment were reported in only 1–2 cases per study (with 17–24 patients included per study, see Table 2), the most common being hypokalemia [13, 65], hypernatremia [13], hypertension and anemia [66]. Riordan et al. [65] additionally reported one case of kidney stones in a metastatic CRC patient with a history of renal calculi, suggesting IVC may be contraindicated for patients with renal dysfunction. Nielsen et al. [66] reported one case of pulmonary embolism and pneumonia each, both of which can also be attributed to the underlying disease, since cancer is known to increase the risk of thromboembolic events. Hoffer at al [12]. reported no grade 3 or higher toxicities.

Beyond being safe and well-tolerated, objective anti-tumor response was not observed in any of these IVC monotherapy studies. While Stephenson et al. [13], Hoffer et al. [12] and Riordan et al. [65] reported 3 (out of 16), 2 (out of 24) and 1 (out of 24), and patients with stable disease, respectively, the study by Nielsen et al. [66] reported no signs of disease remission or stabilization. Latter result is likely related to the fact that both dose and administration frequency (maximum of 60 g whole body dose given 1 time per week for 12 weeks) was considerably lower compared to the other studies (here, up to 3 g/kg were administered at least 3 times per week, for up to 8 weeks, see Table 2). That being said, a number of promising case reports have reported unexpectedly long survival time and in some cases even complete tumour regression of advanced or metastatic disease [67–72]. In future studies, molecular profiling of these exceptional responders would be of high value to explore molecular features that make certain tumors more sensitive to IVC.

Currently, one phase II study is ongoing whereby the effect of high-dose (1.25 g/kg) VitC monotherapy is being studied in resectable or metastatic colorectal, pancreatic and lung tumors (Table 3). The objective of the study is to investigate the effect on pathological tumor response in resectable tumors and to observe objective tumor response in KRAS or BRAF mutant metastatic tumors (NCT03146962) [73]. In addition, one medium-dose effort in bladder cancer (NCT04046094) [74] as well as several oral and/or low-dose monotherapy studies in non-solid tumors (NCT03682029)(NCT03613727)(NCT03964688) [75–77] are currently ongoing in line with the promising pre-clinical data concerning these latter cancer types [21, 78].

In general, high-dose VitC monotherapy has not been clinically assessed in patients that have not received (heavy) prior systemic treatment and that are not terminally ill. This fact may explain the limited response effects observed. Finding a feasible clinical setting to include less heavily pre-treated patients however is complicated, as it would involve denying patients standard of care. For this reason, future applications of high-dose VitC as cancer therapy may rather be in combination strategies and we will focus more on this application in the sections below. However, important lessons regarding administration frequency can be learned from these monotherapy studies, whereby only those studies that administered IVC at least 3 times per week warranted further clinical trials. The recommended doses ranged from 1.5 g/kg [12] to 1.9–2.2 g/kg [13].

### VitC monotherapy in palliative care and quality of life

In palliative care, high-dose VitC is currently gaining ground due to its highly safe and tolerable profile. Not only is high-dose VitC known to relieve pain in cancer patients [79], vast clinical evidence suggests that it has a significant positive impact on patients’ well-being [14, 17–19, 63, 80–83]. This might be due to the frequent hypovitaminosis and VitC deficiency in cancer patients [79, 84, 85], which are commonly enhanced by anti-neoplastic treatments [18].

For instance, a retrospective, multicentre, epidemiological cohort study [18] showed amelioration of appetite, fatigue, depression and sleep disorders in breast cancer and terminal cancer patients suffering from a wide variety of cancer types that received complementary 7.5 g IVC while being treated by respective standard regimens. More recently, a single-center, parallel-group, single-blind interventional study also in breast cancer patients [86] showed a similar and significant reduction of symptoms such as nausea, fatigue, tumor pain and loss of appetite by administering 25 g of IVC per week in addition to their current standard treatment. Favourably, no new side effects were reported after initiation of IVC treatment.

Moreover, another retrospective study showed that patients with radiotherapy-resistant bone metastasis did not only have less pain and better performance measures when given high-dose VitC, they had a median survival time of 10 months as compared to the 2 months median survival time within the control group [80].

Overall, high dose VitC administered as a single agent has not only been shown to be safe and well-tolerated in cancer patients, but also to ameliorate pain and to improve quality of life in the palliative care setting.

## High-dose VitC in combination treatments

Many studies in the past years have investigated high dose VitC as an adjuvant pro-oxidative agent mainly in chemo- and radiotherapy. In addition, other combination treatments have been investigated as well. In this section, we review the pre-clinical and clinical literature of high dose VitC in combination treatments.

**Fig. 1:**
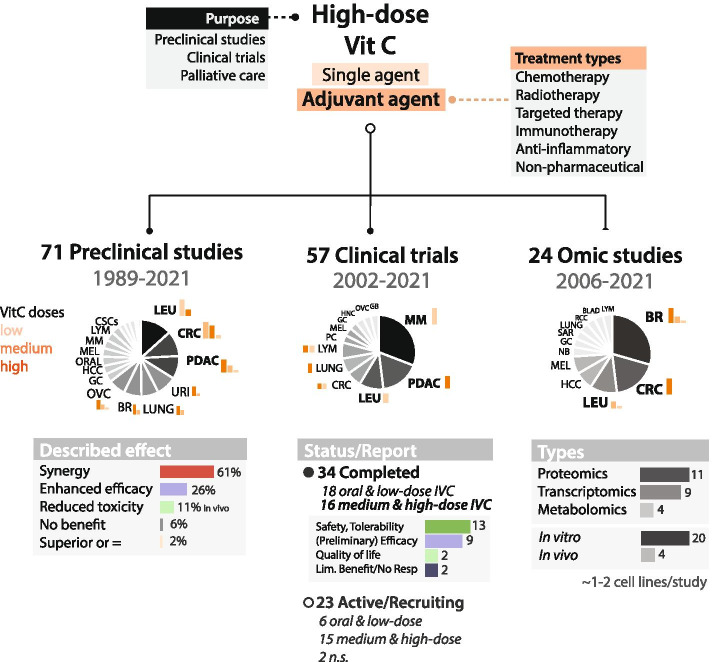
Study overview of pre-clinical, clinical and omics studies using high-dose VitC as anti-cancer agent. Estimated bar graphs of most represented cancer types VitC doses are shown in orange and include high dose (≥ 1 mM in vitro or 1 g/kg in vivo and clinical), medium dose (≤ 0.5 mM in vitro), and low dose (≤ 0.1 mM in vitro,< 1 g/kg in vivo, ≤ 10 g whole body dose clinical). Less represented tumour types are further described in Tables 1, 2, 3 and 4, where oral doses are also included if applicable. Described effect in pre-clinical studies is expressed by percentage of the total number of studies. Reported results in completed clinical trials are expressed by number of studies. Number of studies per global molecular profiling type are also indicated. Omic results include *n* = 20 in vitro and *n* = 4 in vivo studies

**Fig. 2:**
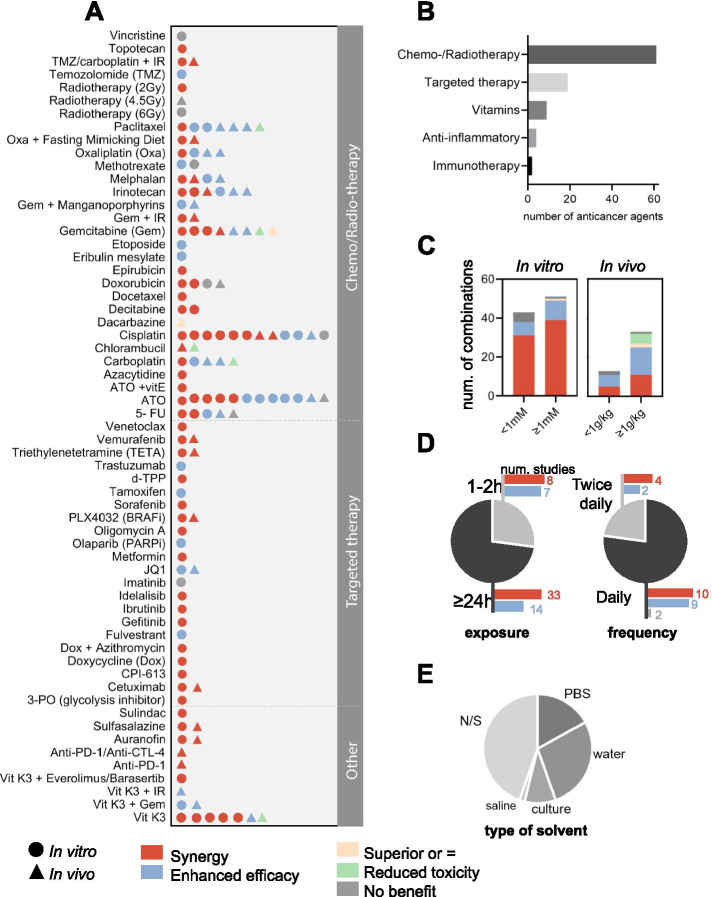
Use of high-dose VitC as adjuvant agent in combination with anti-cancer agents. **A** Described effect of 59 anti-cancer agents combined with high dose vitC investigated in a total of 71 pre-clinical in vitro and in vivo studies (updated may 2021) describing synergy, enhanced efficacy, superior or equivalent effect, reduced toxicity and/or no benefit. **B** Number of combinations per treatment type. **C** Described effect per dose group in vitro and in vivo. **D** Treatment exposure in vitro in hours and frequency dosage in vivo. **E** Described solvent used for VitC preparation. Use of water stands for MiliQ water, demi water and sterile water; N/S, not specified

**Fig. 3:**
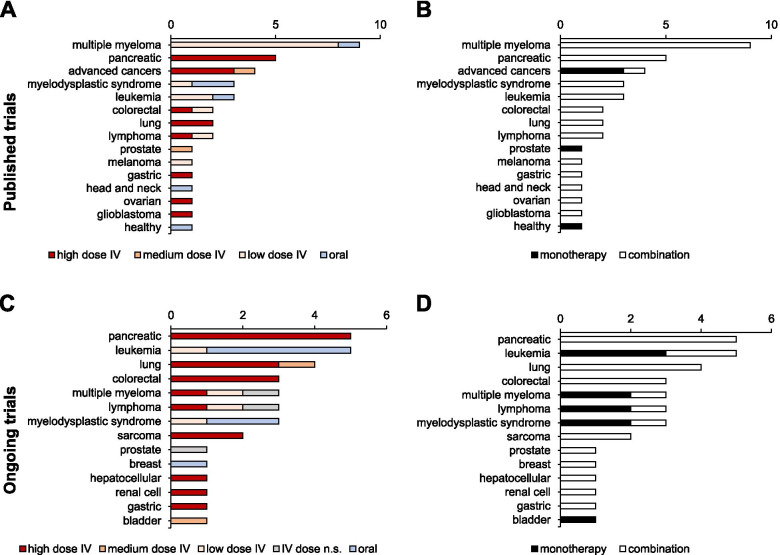
Cancer types investigated in 34 published and 23 ongoing (status February 2021) VitC clinical trials. Annotated are VitC dose group (**A** and **C**; high dose ≥1 g/kg, low dose ≤10 g whole body dose) and treatment type (**B** and **D**). See Table 2 (medium-to-high-dose published trials; 16/34 of total published trials) and Table 3 (medium-to-high dose ongoing trials; 16/23 of total ongoing trials) for details

For pre-clinical studies, we provide detailed information per study and per combination (i.e. cancer type, VitC doses, route of administration, sample size, etc), and describe the observed effects such as synergism, enhanced efficacy and/or reduced toxicity (Table 1, Figs. 1, 2). Particularly for clinical studies, completed and on-going trials using IVC as monotherapy and combination treatment are described in detail (Tables 2, 3, Fig. 3). We examine relevant information on phase of study, type of interventions, IVC dose, injection scheme and number of patients enrolled. In addition, results of completed studies and primary outcomes of ongoing trials are thoroughly discussed.

**Table 1:** Combinations of anti-cancer agents and high-dose VitC in pre-clinical in vitro and in vivo studies

Combination Treatment(s)	Type Drug	Cancer type(s)	***Type of Study***	Sample Size	Dose In vitro	Tx duration	Dose, Administration In vivo	Schedule In vivo	Results	Ref.
2Gy	Radiotherapy	Pancreatic	In vitro	*n* = 1 cell line	4 mM	24 h	–	–	Radio-sensitizing	[87]
5-FU	Chemotherapy	Colorectal	In vitro*,* In vivo	*n* = 3 cell lines, *n* = 48 Balb/c nu/nu mice	0.15–13.3 mM	24, 48, 72, 96 h	150 mg/kg IP	Daily	In vitro synergy, in vivo no benefit	[88]
		Gastric	In vitro*,* In vivo	*n* = 2 cell lines, *n* = 60 athymic-nu/nu mice	1 mM	1 h	4 g/kg IP	Daily (20–30 days)	Enhanced efficacy	[89]
Anti-PD-1	Immunotherapy	B cell lymphoma	In vivo	*n* = 40 immunocompetent syngenic BALB/c mice	–	N/S	1500 mM IP	Daily (dose-escalated, 10-19 days)	Synergy	[90]
Anti-PD-1/Anti-CTL-4	Immunotherapy	Breast, Colorectal, Pancreatic	In vivo	*n* = 13 immunocompetent syngeneic mice	–	N/S	4 g/kg IP	Daily 5x/week	Synergy and effective antitumor immune memory	[91]
ATO	Chemotherapy	Colorectal	In vitro	*n* = 2 cell lines	2 mM	24 h	–	–	Synergy	[92]
		Colorectal, Pancreatic (mKRAS)	In vitro*,* In vivo	*n* = 7 cell lines, *n* = 30 mice	1 mM	48, 72 h	1.5 g/kg IV	Daily	Enhanced efficacy	[93]
		AML and APL	In vitro	*n* = 5 cell lines, *n* = 48 primary cells	3 mM	72 h	–	–	Enhanced efficacy	[94]
		CLL	In vitro	Primary cells of *n* = 18 patients	1 mM	24, 72 h	–	–	Enhanced efficacy	[95]
ATO + vitE	Chemotherapy	APL	In vitro	*n* = 1 cell line	0.1 mM	48 h	–	–	Synergy	[96]
Auranofin	Anti-inflammatory	Triple-Negative Breast	In vitro*,* In vivo	*n* = 5 cell lines, *n* = 25 swiss Nude Mice	2.5 mM	24 h	4 g/kg IP	Daily (15 days)	Synergy	[97]
Azacytidine	Chemotherapy	Colorectal	In vitro	*n* = 1 cell line	0.01, 0.05 mM	72 h	–	–	Synergy	[98]
Carboplatin	Chemotherapy	Gastric	In vitro*,* In vivo	*n* = 2 cell line, *n* = 60 athymic-nu/nu mice	1 mM	1 h	4 g/kg IP	Daily (20–30 days)	Enhanced efficacy	[89]
Cetuximab	Targeted therapy	Colorectal (mKRAS)	In vitro*,* In vivo	*n* = 5 cell lines, *n* = N/S athymic nude mice	0.3, 0.5, 0.7 mM	6 h	0.5 g/kg IP	Daily (14 days)	Synergy and abrogates resistance via SVCT-2	[99]
Cisplatin	Chemotherapy	Gastric	In vitro	*n* = 1 cell line	0.000284, 0.000568 mM	48 h	–	–	Synergy	[100]
		Cervical	In vitro	*n* = 2 cell lines	0.000568 mM	24, 48, 72 h	–	–	Synergy	[101]
		Oral squamous	In vitro*,* In vivo	*n* = 8 cell lines, *n* = 24 C57BL/6 mice	0.125, 0.25, 0.5, 1 mM	72 h	4 g/kg IP	Daily (21 days)	Synergy	[51]
		Ovarian	In vitro	*n* = 1 cell line	2 mM	2 h	–	–	Enhanced efficacy	[102]
		Cervical	In vitro	*n* = 2 cell lines	1, 2.5, 3.3, 16 mM	24, 48, 72 h	–	–	Synergy	[103]
		Gastric	In vitro*,* In vivo	*n* = 2 cell lines, *n* = 60 athymic-nu/nu mice	1 mM	1 h	4 g/kg IP	Daily (20–30 days)	Enhanced efficacy	[89]
CPI-613	Targeted therapy	CLL	In vitro	*n* = 2 cell lines	0.1–2 mM	24 h	–	–	Synergy	[104]
Decitabine	Chemotherapy	AML	In vitro	*n* = 2 cell lines	0.3 mM	24, 48, 72 h	–	–	Synergy	[105]
		Colorectal	In vitro	*n* = 1 cells line	0.01, 0.05 mM	72 h	–	–	Synergy	[98]
Doxorubicin	Chemotherapy	Cervical	In vitro	*n* = 2 cell lines	1, 2.5, 3.3, 16 mM	24, 48, 72 h	–	–	Synergy	[103]
Doxycycline	Targeted therapy	Cancer Stem Cells	In vitro	*n* = 1 cells line	0.25–0.5 mM	5 days	–	–	Synergy	[106]
Doxycycline + Azithromycin	Targeted therapy	Cancer Stem Cells	In vitro	*n* = 1 cell line	0.25 mM	5 days	–	–	Synergy	[107]
Eribulin mesylate	Chemotherapy	Breast	In vitro	*n* = 6 cell lines	5, 10, 20 mM	2 h (×1 or ×2)	–	–	Enhanced efficacy	[108]
Etoposide	Chemotherapy	Glioblastoma	In vitro	*n* = 1 cell line	1 mM	48, 96, 144 h	–	–	Enhanced efficacy	[54]
Fulvestrant	Hormonal therapy	Breast	In vitro	*n* = 6 cell lines	5, 10, 20 mM	2 h (×1 or ×2)	–	–	Enhanced efficacy	[108]
Gefitinib	Targeted therapy	Non-small cell Lung	In vitro	*n* = 3 cell lines	0.5, 1, 2.5, 5, 10 mM	1 h	–	–	Synergy	[109]
Gemcitabine	Chemotherapy	Pancreatic	In vitro*,* In vivo	*n* = 6 cell lines, *n* = N/S athymic nude mice	0.001 mM	1 h	4 g/kg IP	Twice daily (6 days)	Radioprotection and radiosensitization	[110]
		Pancreatic	In vivo	*n* = 32 mice	–	–	4 g/kg IP	Daily (45 days)	Enhanced efficacy and VitC equal to combination	[14]
Gemcitabine + Ionizing radiation (IR)	Chemoradiotherapy	Sarcoma	In vitro*,* In vivo	*n* = 2 cell lines, *n* ≥ 7 per treatment group, athymic-nu/nu mice	2, 5 mM	1 h	4 g/kg IP	Daily (40-60 days)	Radio-chemo sensitizer	[111]
Ibrutinib	Targeted therapy	CLL	In vitro	*n* = 2 cell lines, *n* = 6 primary cells	0.1–2 mM	24 h	–	–	Synergy	[104]
Idelalisib	Targeted therapy	CLL	In vitro	*n* = 2 cell lines, primary cells of *n* = 6 patients	0.1–2 mM	24 h	–	–	Synergy	[104]
Irinotecan	Chemotherapy	Colorectal	In vitro*,* In vivo	*n* = 3 cell lines, *n* = 48 Balb/c nu/nu mice	0.15–13.3 mM	24, 48, 72, 96 h	150 mg/kg IP	Daily	Synergy in vitro, enhanced efficacy in vivo	[88]
		Gastric	In vitro*,* In vivo	*n* = 2 cell lines, *n* = 60 athymic-nu/nu mice	1 mM	1 h	4 g/kg IP	Daily (20–30 days)	Enhanced efficacy	[89]
		Gastric	In vitro*,* In vivo	*n* = 5 cell lines, *n* = 24 ALB/c nude mice	2, 4 mM	2 h	4 g/kg IP	Twice daily	Synergy	[112]
Melphalan	Chemotherapy	Multiple Myeloma	In vitro*,* In vivo	Primary cells of *n* = 13 patients, *n* = 45 NOD.Cγ-Rag1 mice	8, 20 mM	1 h	4 mg/kg IP	Daily	Synergy	[113]
Metformin	Multitargeted Therapy	CLL	In vitro	*n* = 2 cell lines	0.1–2 mM	24 h	–	–	Synergy	[104]
Olaparib (PARP inhibitor)	Targeted therapy	AML (TET2-deficient)	In vitro	*n* = 6 cell lines	0.125, 0.25, 0.5, 1 mM	72 h	–	–	Enhanced sensitivity	[22]
Oligomycin A	Targeted therapy	CLL	In vitro	*n* = 2 cell lines	0.1–2 mM	24 h	–	–	Synergy	[104]
Oxaliplatin	Chemotherapy	Colorectal	In vitro*,* In vivo	*n* = 3 cell lines, *n* = 48 (6 × 8) Balb/c nu/nu mice	0.15–13.3 mM	24, 48, 72, 96 h	150 mg/kg IP	Daily	Synergy in vitro, enhanced efficacy in vivo	[88]
		Gastric	In vitro*,* In vivo	*n* = 5 cell lines, *n* = 24 ALB/c nude mice	2, 4 mM	2 h	4 g/kg IP	Twice daily	Synergy in vitro, enhanced efficacy in vivo	[112]
Oxaliplatin + Fasting mimicking diet (FMD)	Chemotherapy + Fasting	Colorectal, Pancreatic, Lung (mKRAS); Prostate, Ovarian	In vitro*,* In vivo	*n* = 11 cell lines, *n* = 38 NSG and BALB/c mice	≥0.3 mM	24 h	4 g/kg IP	Twice daily (36 days)	Synergy	[114]
Paclitaxel	Chemotherapy	Oral squamous	In vivo	*n* = 96 Swiss albino mice	–	N/S	10 mg oral	–	Enhanced efficacy	[115]
		Gastric	In vitro*,* In vivo	*n* = 2 cell lines, *n* = 60 athymic-nu/nu mice	1 mM	1 h	4 g/kg IP	Daily (20–30 days)	Enhanced efficacy	[89]
PLX4032	Targeted therapy	Thyroid	In vitro*,* In vivo	*n* = 3 cell lines; *n* = 20 nude mice	0.1–2 mM	72 h	3 g/kg IP	Daily (15 days)	Synergy	[64]
Sorafenib	Targeted therapy	Liver	In vitro	*n* = 5 cell lines	2.5, 5, 7.5, 10, 20 mM	2 h	–	–	Synergy	[116]
Sulfasalazine	Anti-inflammatory	Prostate	In vitro*,* In vivo	*n* = 2 cell lines, *n* = ~ 24 BALB/c nude mice	1, 2 mM	2-48 h	4 g/kg IP	Twice daily (16 days)	Synergy	[117]
Sulindac	Anti-inflammatory	Colorectal	In vitro	*n* = 2 cell lines	0.5 mM	48 h	–	–	Synergy	[118]
Tamoxifen	Hormonal therapy	Breast	In vitro	*n* = 6 cell lines	5, 10, 20 mM	2 h (×1 or ×2)	–	–	Enhanced efficacy	[108]
Temozolomide	Chemotherapy	Glioblastoma	In vitro	*n* = 1 cell line	1 mM	48, 96, 144 h	–	–	Enhanced efficacy	[54]
Thieno-triazolo-1,4-diazepine (JQ1)	Targeted therapy	Melanoma	In vitro*,* In vivo	*n* = 5 cell lines; *n* = 10 Gulo−/− and 10 Gulo+/+ mice	0.00005–0.0001 mM	72 h	3.3 g/L and 0.33 g/L, oral	Daily (14 days)	Enhanced efficacy	[119]
TMZ/carboplatin + IR	Chemoradiotherapy	Glioblastoma, Non-small cell Lung	In vitro*,* In vivo	*n* = 12 cell lines, *n* = ~ 42 athymic nude mice	1, 2 mM	1 h	4 g/kg IP	Daily	Radio-chemo sensitizer	[16]
Topotecan	Chemotherapy	Breast	In vitro	*n* = 1 cell line	1 mM	48 h	–	–	Synergy	[120]
TPP derivative dodecyl-TPP (d-TPP)	Targeted therapy	Cancer Stem Cells	In vitro	*n* = 2 cell lines	0.25–0.5 mM	5 days	–	–	Synergy	[121]
Trastuzumab	Targeted therapy	Breast	In vitro	*n* = 6 cell lines	5, 10, 20 mM	2 h (×1 or ×2)	–	–	Enhanced efficacy	[108]
Triethylenetetramine (TETA)	Targeted therapy	Breast	In vitro*,* In vivo	*n* = 9 cell lines, *n* = 40 BALB/c-nu	1 mM	12, 24 h	3 g/kg IP	Daily (25 days)	Synergy	[122]
Vemurafenib	Targeted therapy	BRAF mutant Melanoma	In vitro*,* In vivo	*n* = 2 cell lines, *n* = 18 C57BL/6 mice	1, 5 mM	48 h	0.03 mg/kg oral	Daily	Synergy and abrogates resistance	[123]
Venetoclax	Targeted therapy	CLL	In vitro	*n* = 2 cell lines, primary cells of *n* = 6 patients	0.1–2 mM	24 h	–	–	Synergy	[104]
Vit K3 (Menadione) + Everolimus or Barasertib	Vitamin + Targeted therapy	ALL	In vitro	*n* = 1 cell line	0.3 mM	24, 72 h	–	–	Synergy	[124]

A total of 47 combinations in 44 pre-clinical studies from 2016 to 2021 were retrieved from PubMed using search terms (vitamin c OR ascorbate OR ascorbic acid) AND (combination OR synergy OR combined) AND (cancer)

*Tx* treatment, *mM* millimolar, *IP* intraperitoneal, *IV* intravenous, *JQ1* Thieno-triazolo-1,4-diazepine, *5-FU* 5-fluorouracil, *Vit* vitamin, *IR* irradiation, *TMZ* temozolomide, *Gem* gemcitabine, *Dox* Doxycycline, *Oxa* oxaplatin, *TETA* Triethylenetetramine, *BRAF* v-raf murine sarcoma viral oncogene homolog B1, *PARP* poly (ADP-ribose) polymerase, *d-TPP* TPP derivative dodecyl-TPP, *ATO* arsenic trioxide, *3-PO* 3-(3-Pyridinyl)-1-(4-pyridinyl)-2-propen-1-one, *CLL* chronic lymphocytic leukemia, *AML* acute myeloid leukemia, *APL* acute promyelocytic leukemia, *ALL* acute lymphoblastic leukemia, *TET* ten eleven translocation

**Table 2:** 16 published clinical studies using medium-to-high dose IVC as anti-cancer therapy

Cancer type (s)	Allocation/Phase	Interventions	VitC IV dose^a^	VitC dosage and injection scheme	No. patients	Results	Conclusions/Comment	Ref.
IVC monotherapy								
Advanced cancers	Single group, Phase 1	IVC monotherapy	high	30–110 g/m2 (0.8–3.0 g/kg), 4x/week, 4 weeks (both consecutive), rate of 1 g/min	17	All doses were well tolerated. Doses of 70, 90, and 110 g/m2 maintained levels at or above 10–20 mM for 5–6 h (Cmax 49 mM). No objective antitumor response	Recommended dose for future studies is 70–80 g/m2 (= 1.9–2.2 g/kg) based on Cmax	[13, 125]
	Single group, Phase 1	IVC monotherapy	high	0.4–1.5 g/kg, 3x/week, 4 week treatment cycles; oral dose of 500 mg twice daily on non-infusion days	24	Well tolerated, without significant toxicity; dose of 1.5 g/kg sustains plasma ascorbic acid concentrations > 10 mM for > 4 h (Cmax 26 mM); 2 patients with unexpected stable disease	The recommended phase 2 dose is 1.5 g/kg; ascorbate may need to be combined with cytotoxic or other redoxactive molecules to be an efficacious treatment	[12], no ClinicalTrial.gov Identifier
	Single group, Phase *n.s.*	IVC monotherapy	medium	0.15–0.71 g/kg/day, continuous infusion for up to 8 weeks	24	IVC therapy relatively safe, only few and minor adverse events observed; plasma ascorbate concentrations in the order of 1 mM attained	Further clinical studies with high dose IVC are warranted	[65], no ClinicalTrial.gov Identifier
Prostate	Phase 2	IVC monotherapy	medium	5 g week 1, 30 g week 2 and 60 g weeks 3–12; daily oral dose of 500 mg starting after first infusion for 26 weeks	23	No patient achieved the primary endpoint of 50% PSA reduction; instead, a median increase in PSA of 17 μg/L was recorded at week 12; no signs of disease remission were observed; target dose of 60 g AA IV produced a peak plasma AA concentration of 20.3 mM [126]	This study does not support the use of intravenous AA outside clinical trials	[66, 126, 127]
IVC combination therapy - Chemotherapy and radiation therapy								
Advanced cancers	Single group, Phase 1/2	IVC + standard care cytotoxic chemotherapy	high	1.5 g/kg, 2 or 3x per week	14	IVC-chemotherapy is non-toxic and generally well tolerated; individual highly favourable responses found in biliary tract, cervix and head and neck cancer patients, colorectal cancer patients without benefit	Neither proves nor disproves IVC’s value in cancer therapy; illustrates potential for “discovery in clinical practice”	[83, 128]
Glioblastoma	Single group, Phase 1	IVC + RT + temozolomide (TMZ)	high	Radiation phase: 15–125 g, 3x weekly, 7 weeks; Adjuvant phase: dose-escalation until plasma level of 20 mM was achieved, 2x weekly, 28 weeks	13	Safe and well tolerated; targeted ascorbate plasma levels of 20 mmol/L achieved in the 87.5 g cohort; favourable OS and PFS compared to historical controls (RT + TMZ only)	Phase 2 clinical trial initiated (NCT02344355), currently active, not recruiting	[16, 129, 130]
NSCLC	Single group, Phase 2	IVC + carboplatin + paclitaxel	high	75 g, 2x weekly	14	Increased disease control and objective response rates	Still recruiting (NCT02420314), see Table 3	[16, 133]
Ovarian	Phase 1/2a, randomized	Arm 1: IVC + carboplatin + paclitaxel Arm 2: carboplatin + paclitaxel only	high	Dose escalation up to 75 or 100 g, with target peak plasma concentration of 350 to 400 mg/dl (20 to 23 mM), 2x/week, for 12 months (of which the first 6 months in conjunction with chemotherapy)	25	Longer PFS and substantially decreased toxicities compared to control arm w/o Vit C; trend toward improved median OS	Study not powered for detection of efficacy, larger clinical trials warranted	[63, 145]
Pancreatic	Single group, Phase 1/2a	IVC + gemcitabine	high	25–100 g dose escalation in phase I, 75–100 g in phase II, 3x weekly, for 4 weeks	14	Well tolerated, no clinically significant influence on gemcitabine pharmacokinetics	Phase 2/3 trial needed to detect efficacy and benefit of IVC	[14, 146]
	Single group, Phase 1	IVC + RT + gemcitabine	high	50–100 g daily during RT, 6 weeks	16	Safe and well tolerated with suggestions of efficacy; increased OS and PFS compared to institutional average; 100 g determined to be MTD, 75 g selected as a recommended phase II dose	Phase 2 trial is indicated	[110, 147]
	Phase 2, randomized	Arm 1: IVC + G-FLIP/G-FLIP-DM Arm 2: G-FLIP/G-FLIP-DM only	high	75–100 g, 1–2x per week, with GFLIP every every 2 weeks until progression	26	Safe and well tolerated. May avoid standard 20–40% rates of severe toxicities	Abstract only, no data shown	[148, 149]
	Single group, Phase 1	IVC + gemcitabine	high	50–125 g, 2x weekly to achieve target plasma level of ≥350 mg/dL (≥20 mM)	9	Well-tolerated with suggestion of some efficacy; plasma levels of 20–30 mM were reached with doses ranging from 0.75–1.75 g/kg	Phase 2 trial is indicated	[82, 150]
IVC combination therapy - Targeted therapy								
Colorectal, Gastric	Single group, Phase 1	IVC + mFOLFOX6 or FOLFIRI (part 1); IVC + mFOLFOX6 ± bevacizumab (part 2)	high	Dose escalation phase (part 1): 0.2–1.5 g/kg, once daily, days 1–3, in a 14-day cycle until MTD was reached; Speed expansion phase (part 2): MTD or at 1.5 g/kg if MTD not reached	36 (30 colorectal, 6 gastric)	MTD not reached; no DLT; favourable safety profile and preliminary efficacy	Recommended dose for future studies 1.5 g/kg/day; extended to phase 3 study	[151, 152]
Pancreatic	Single group, Phase 1	IVC + gemcitabine + erlotinib	high	50–100 g, 3x/week, 8 weeks	9	Tumor shrinkage in 8/9 patients; peak ascorbic acid concentrations as high as 30 mmol/L in the highest dose group	Phase 2 trial with longer treatment period 100 g dosage warranted	[153, 154]
B-cell non-Hodgkin’s lymphoma	Single group, Phase 1	IVC + CHASER regimen	high	75 g or 100 g 5x in 3 weeks	3	Whole body dose of 75 g safe and sufficient to achieve an effective serum concentration (> 15 mM (264 mg/dl)	No NCT number; Phase II trial is indicated	[155], no ClinicalTrial.gov Identifier
IVC combination therapy - Combinations with emerging non-pharmaceutical therapies								
NSCLC	Phase 1/2, randomized	Arm 1: IVC + mEHT + BSC Arm 2: BSC alone	high	1 g/kg, 1.2 g/kg or 1.5 g/kg, 3x/week for 8 weeks (Phase 1); 1 g/kg, 3x/week, 25 treatments in total (Phase 2)	97	IVC treatment concurrent with mEHT is safe and improved the QoL of NSCLC patients (Phase 1, Ou et al., 2017); significantly prolonged PFS, OS and QoL (Phase 2)	IVC + mEHT is a feasible treatment in advanced NSCLC	[156–158]

Shown are the 16 published trials using medium-to-high dose IVC out of a total 34 published trials. All 34 trials, including those using low-dose or oral VitC, are summarized in Fig. 3. Entries are ordered primarily by kind of combination treatment, and secondarily by cancer type

^a^High dose ≥1 g/kg, low dose ≤10 g whole body dose

n.s., not specified; g/kg × 37 = g/m2 (1.5 g/kg = 56 g/m2); G-FLIP/G-FLIP-DM: low dose Gemcitabine, fluorouracil, leucovorin, irinotecan, and oxaliplatin/ G-FLIP + low dose docetaxel and mitomycin C; CHASER regimen: Rituximab, cyclophosphamide, cytarabine, etoposide and dexamethasone; mFOLFOX6/FOLFIRI, oxaliplatin, leucovorin and 5-fluorouracil/irinotecan, leucovorin and 5-fluorouracil

**Table 3:** 16 ongoing clincal studies using medium-to-high dose IVC as anti-cancer therapy

Cancer type(s)	NCT Number	Allocation/ Phase	Interventions	Type of combination therapy	VitC IV dose*	VitC dose and administration schedule	Estimated enrollment	Primary outcome(s)
Colorectal	NCT04516681 [131]	Randomized, Phase 3	Arm 1: Ascorbic acid + chemotherapy Arm 2: Chemotherapy alone (FOLFOXIRI+/− bevacizumab)	Chemo + Targeted	high	1.5 g/kg/day, D1–3, every 2 weeks	400	Objective Response Rate
Colorectal, Pancreatic, Lung	NCT03146962 [73]	Single group, Phase 2	Cohort A: VitC for 2–4 consecutive weeks Cohort 2: VitC up to 6 months Cohort 3: VitC for 1–2 weeks prior to and following Y90 radioembolization of hepatic metastases	RE	high	1.25 g/kg for 4 days/week	50	Pathologic response (cohort A) 3-month disease control rate (DCR) (cohort B) Maximal tolerated dose (cohort C)
Hepatocellular, Pancreatic, Gastric, Colorectal	NCT04033107 [132]	Single group, Phase 2	VitC + metformin	Targeted	high	1.5 g/kg, D1–3, every 2 weeks	30	Progression-free survival
Lung	NCT02420314 [133]	Single group, Phase 2	Ascorbic acid + paclitaxel + carboplatin	Chemo	high	75 g, two times/week	57	Tumor response
Lung	NCT02905591 [134]	Single group, Phase 2	Ascorbate + chemoRT (radiation therapy + paclitaxel + carboplatin)	Chemo-RT	high	75 g, 3 times/week	46	Progression rate
Lymphoma	NCT03602235 [135]	Single group, Phase 1	VitC + melphalan	Chemo	high	50 g, 75 g and 100 g (3 + 3 cohort method)	9	Number of treatment related adverse events
Lymphoma	NCT03418038 [136]	Randomized, Phase 2	Arm 1: Ascorbic acid + combination chemotherapy Arm 2: Placebo + combination chemotherapy (rituximab + ifosfamide + carboplatin + etoposide D1–3; rituximab + cisplatin + cytarabine + dexamethasone if MR or SD after 2 courses) Arm 3: Ascorbic acid + combination chemotherapy (ifosfamide + carboplatin + etoposide or cisplatin + cytarabine + dexamethasone or gemcitabine + dexamethasone + cisplatin or gemcitabine + oxaliplatin or oxaliplatin + cytarabine + dexamethasone)	Chemo + Targeted + Corticosteroid	high	High dose (*n.s.*) on D1, 3, 5, 8, 10, 12, 15, 17 and 19, combination chemotherapy on D1–3; treatment repeats every 21 days for up to 4 courses	151	Overall response rate
Pancreatic	NCT02905578 [137]	Randomized, Phase 2	Arm 1: Ascorbate + chemotherapy Arm 2: Chemotherapy alone (gemcitabine + nab-paclitaxel)	Chemo	high	75 g, three times/weekly for 4 weeks	65	Overall survival
Pancreatic	NCT04150042 [138]	Single group, Phase 1	VitC + chemotherapy/stem cell treatment (melphalan + carmustine + vitamin B12B + ethanol)	Chemo + Dietary suppl.	high	Dose-escalation beginning with 3 g/m^2 and escalating to a maximum of 8 g/m^2	10	Rate of mucositis, rate of engraftment of Neutrophils + adverse events, among others
Pancreatic	NCT03410030 [139]	Single group, Phase 1/2	Ascorbic acid + nab-paclitaxel + cisplatin + gemcitabine	Chemo	high	≥ 20 mM plasma concentration	36	Phase IB: recommended phase II dose (to reach ≥20 mM) Phase II: disease control rate
Prostate	NCT02516670 [140]	Randomized, Phase 2	Arm 1: Ascorbate + Docetaxel Arm 2: Placebo + Docetaxel	Chemo	high	1 g/kg, 3 times/ week	69	Occurrence of PSA decline of > = 50% + adverse events
Renal Cell	NCT03334409 [141]	Randomized, Phase 2	Arm 1: Ascorbic acid + tyrosine kinase inhibitor Arm 2: Tyrosine kinase inhibitor alone (Pazopanib)	Targeted	high	1 g/kg 3 times/week	91	Treatment failure-free rate
Sarcoma	NCT04634227 [142]	Single group, Early phase 1	Ascorbate + gemcitabine	Chemo	high	75 g dose on D1–2, until target serum concentration between 20 and 30 mM (otherwise maximum dose of 125 g)	20	Progression-free survival
Sarcoma	NCT03508726 [143]	Single group, Phase 1/2	Ascorbate + radiation therapy	RT	high	75 g, three times/week	25	Incidence of dose limiting toxicities (DLTs) + tumor response
Bladder	NCT04046094 [74]	Single group, Phase 1/2	Ascorbic acid	–	medium	25 g, 2 times/week for 4 weeks	21	Post treatment pathological staging
Lung	NCT03799094 [144]	Randomized, Phase 1/2	Arm 1: VitC + tyrosine kinase inhibitor Arm 2: Tyrosine kinase inhibitor alone (osimertinib, erlotinib or gefitinib)	Targeted	medium	30 g once/week	150	Progression-free survival

Shown are the 16 trials using medium-to-high dose IVC out of a total 23 studies currently recruiting (status February 2021), as retrieved from the *clinicaltrials.gov**database* (see also Fig. 3). Entries are ordered primarily by high-to-medium IVC dose, and secondarily by cancer type

### Pre-clinical combination studies

A comprehensive overview of all 71 retrieved studies from 1989 to 2021 (Fig. 1), investigating 59 combinations, is shown in Fig. 2, while the 44 studies of the last 5 years are summarized in more detail in Table 1. A division can be made between the highly studied combination with chemotherapy and radiotherapy, the lesser studied with targeted therapies, combinations with immune therapy, which has only more recently gained awareness, and with non-conventional anti-cancer agents (Fig. 2B).

#### Pre-clinical studies using VitC in combination with chemotherapy and radiation therapy

In pre-clinical models, high-dose VitC is reported to enhance the effectivity of a wide variety of chemotherapeutics such as carboplatin [63, 89], cisplatin [51, 89, 100–103, 179, 180], chlorambucil [181], 5-FU [88, 89, 182], gemcitabine [14, 110, 183, 184] and temozolomide [16, 54] in various cancer cell types, often in a synergistic manner or by enhancing treatment efficacy (Table 1 and Fig. 2).

For example, a recent in vivo study in oral squamous carcinoma described an enhanced therapeutic effect of cisplatin in combination with high-dose VitC (4 g/kg IP twice daily) [51]. A study in pancreatic cancer showed that gemcitabine given in combination with high-dose VitC (4 g/kg IP twice daily) achieved significant tumor growth inhibition in mice bearing pancreatic xenografts compared to control and gemcitabine-only groups [14].

Similarly promising, high-dose VitC has also been found to act as a radio-sensitizer during radiation or chemo-radiation of pre-clinical cancer models, with high specificity for cancer cells over healthy cells [16, 87, 89, 110, 111, 185–190].

A notable example is the study of Schoenfeld et al. [16], which investigated combinations of standard cisplatin chemotherapy with VitC in NSCLC and standard temozolomide and radiation in GBM. To this end, they studied cell line models, performed in vivo studies and a phase I/II clinical trial. Mice injected with high-dose VitC (4 g/kg IP daily) in combination with radio-chemotherapy (5 mg/kg carboplatin weekly, 12 Gy IR/2 fractions (fx)) significantly increased overall survival (~ 50% increase), sensitizing these hard-to-treat NSCLC and GMB tumours to current treatment regimens. Similar results in gastric cancer were described by O’Leary et al., whereby high-dose VitC (4 g/kg IP daily) was injected in combination with carboplatin (15 mg/kg weekly), paclitaxel (10 mg/kg) and 2Gy IR/8fx [89]. An important consideration for pre-clinical combination studies is the clinical standard of care onto which VitC is added, as exemplified by a study in GBM [191] that demonstrated faster tumor progression in tumor-bearing mice treated with a single dose of radiation and daily high-dose ascorbate than in those treated with radiation alone. Here, the authors use a single 4.5 Gy irradiation dose, which does not relate to standard treatment of care in GBM patients who receive daily fractions up to a total of 60 Gy. In addition, the relatively seen lower ascorbate dose of 1 or 2 g/kg compared to the 4 g/kg applied in the GBM study by Schoenfeld et al. [16], possibly promoted VitCs radio-protective rather than radio-sensitizing properties.

Finally, in addition to its enhancing effects in conventional cytotoxic therapies, numerous animal studies have shown decreased off-target toxicity of (chemo-) therapeutic agents following administration of OC and IVC [192]. In this review, Carr and Cook reported that VitC administration typically decreases white blood cell loss, weight loss, ascites accumulation, hepatotoxicity, reticulocytosis, lipid oxidation and cardiomyopathy induced by the chemotherapeutic agents.

#### Pre-clinical studies using VitC in combination with targeted therapy

A great number of pre-clinical studies have examined the use of high-dose VitC combined with targeted therapies such as kinase inhibitors (i.e. sorafenib, gefitinib, vemurafenib) [109, 116, 123], mitochondrial inhibitors (i.e. doxycycline, venetoclax, oligomycin A, metformin) [104, 106, 107], poly ADP ribose polymerase (PARP) inhibitors [193] and glycolysis inhibitors [194].

Overall, most of the retrieved pre-clinical studies reported synergistic effects in vitro and/or in vivo (Fig. 2A), warranting clinical studies*.* For instance, an in vitro study showed synergistic anti-cancer action of high-dose VitC in combination with sorafenib, a multi-kinase (eg. Raf-1, B-Raf, VEGFR-1-3 and FLT3) inhibitor, in hepatocellular carcinoma (HCC) cells, and additionally reported a case of prolonged regression of a HCC patient upon combination treatment with IV high-dose VitC and sorafenib [116]. Other studies have reported similar synergistic effects for high-dose VitC combined with EGFR inhibitors cetuximab and gefitinib in KRAS mutated colon cancer and NSCLC cells respectively [99, 109]. Interestingly, Jung et al. [99] showed that medium-dose VitC (0.5 g kg^− 1^) could abrogate cetuximab resistance in vivo and suggested sodium-dependent vitamin C transporter SVCT2 as a potental marker for enhancing efficacy of the combination treatment of VitC and cetuximab in KRAS-mutant CRC patients. Similarly, resistance to BRAFV600 inhibitor vemurafenib was also abrogated by VitC in melanoma in vivo [123]. Recent findings reinforce the promising synergistic effects of VitC with kinase inhibitors such as BRAFV600 inhibitor PLX4032 in thyroid cancer in vivo [64] and with BTK inhibitor ibrutinib and PI3K inhibitor idelalisib in chronic lymphocytic leukemia (CLL) patient-derived cells [104].

Likewise, emerging anti-cancer compounds targeting telomerases, mitochondrial activity or glycolysis also synergize with high-dose VitC. For instance, telomerase inhibitor triethylenetetramine (TETA) in the treatment of breast cancer [122], glycolysis inhibitor 3-(3-Pyridinyl)-1-(4-pyridinyl)-2-propen-1-one (3-PO) in NSCLC cells [194], respiratory chain complex I inhibitor metformin, ATP synthase inhibitor oligomycin A and Bcl-2 inhibitor venetoclax in CLL patient-derived cells [104].

Furthermore, enhanced treatment efficacy was confirmed for high-dose VitC in combination with several hormonal treatments such as oestrogen receptor ER and human epidermal growth factor receptor 2 (HER2) inhibitors in breast cancer cells [108], as well as for PARP inhibition in the treatment of AML-TET2 deficient cells [22] and JQ1 (thieno-triazolo-1,4-diazepine), a Bromodomain and extraterminal inhibitor, in the treatment of melanoma [36].

Finally, three recent in vitro studies indicate that high-dose VitC might be of use in eradicating cancer stem cells (CSC) by synergistically targeting mitochondria and causing cell death combined with several targeted agents [106, 107, 121].

All data combined strongly emphasizes the potential of high-dose VitC as adjuvant therapy for targeted therapies.

#### Pre-clinical studies using VitC in combination with immunotherapy and anti-inflammatory compounds

Little research has been conducted on high-dose VitC in combination with immunotherapy. Two very recent studies show that high-dose VitC synergizes with immune checkpoint inhibitors (ICI) anti-PD-1 and anti-CTL-4 in mouse models, as well as increases the immunogenicity of effector T cells [90, 91]. For instance, Luchtel et al. [90] pre-treated lymphoma cells co-cultured with CD8+ T cells derived from healthy donors with 1 mM VitC. Interestingly, they described a significant 15–21% increase in immunogenicity compared to non-VitC treated cells.

In combination with ICI, high-dose VitC affected tumour growth in a T cell–dependent manner, by attracting effector T-cells and not T regulatory cells. Importantly, in a few mice, complete regressions were observed and mice also acquired immunity after re-injection of tumour cells [91]. Of note, mismatch repair deficient tumours, usually resistant to ICI, showed a very effective response when combined with high-dose VitC. In addition, upon high-dose VitC administration, not only CD8+ T cells, but also macrophages showed increased tumour infiltration, and both enhanced Granzyme B production by cytotoxic T cells and enhanced interleukin 12 production by antigen-presenting cells were observed. These studies are particularly encouraging given the great potential of immunotherapy in anti-cancer treatment, and suggest that high-dose VitC may be a promising combination strategy to convert “cold” tumours into “hot” tumours, further widening the therapeutic scope of immunotherapy.

Furthermore, high-dose VitC strongly enhanced anti-cancer effects of immunosuppressor auranofin in the treatment of triple-negative breast cancer in vitro and in vivo [97]. Similarly, anti-inflammatory compounds such as sulindac [118], sulfasalazine [117] and methotrexate [195] showed strong synergy and enhanced efficacy in the treatment of colon, prostate and liver cancer, respectively.

#### Pre-clinical studies using VitC in combination with emerging non-pharmaceutical therapies

High-dose VitC has also been combined with other less conventional regimens. One study reports the synergistic effect of fasting-mimicking diet and oxaliplatin in combination with high-dose VitC against KRAS mutated cancers both in vitro and in vivo [114].

In addition, several studies reported synergism of anti-cancer effects of vitamin K3, also known as menadione, combined with VitC in vitro [21, 196–199]. Moreover, one in vivo study found that the combination of these vitamins reduced tumor growth and tumor metastasis in Lewis lung carcinoma [59]. In addition, this vitamin combination was also reported to be synergistic with mTOR inhibitor everolimus and aurora B kinase inhibitor barasertib [124] and sensitized human urothelial tumors to gemcitabine [200] and various solid tumors to radiotherapy in vivo [201], mainly causing cell death upon oxidative stress [202].

#### Technical considerations and need for standardization

To deduce best practices, we further evaluated dosing schedules, duration of treatment and solvents used in the pre-clinical studies (Table 1, Fig. 2B-E).

First, the type of solvent used for preparing VitC solutions significantly varies, water being the preferred one, followed by phosphate-buffered saline (PBS), culture media -for in vitro studies- and saline -for in vivo studies- (Fig. 2E). Notably, almost 45% of studies did not report the type of solvent used in their methods section. Likewise, most of the studies did not indicate the use of seal to prevent oxygen and light interaction, nor pH range used. In light of VitC chemistry and stability, these are important considerations that should be standardized to get reproducible and robust results [16, 203, 204].

Since VitC effect is dose-dependent, we examined the effect among different dose groups, ≥1 mM vs. < 1 mM in vitro and ≥ 1 g/kg vs. < 1 g/kg in vivo (Fig. 2C). For in vitro studies, a synergistic effect was reported in 80% of all cases and 20% showed enhanced efficacy. Given that 2D and 3D cell culture cannot fully reproduce physiological conditions, in vivo studies provide added value for clinical studies. For in vivo IP injections, synergism was reported two times more often in the studies that used a higher dose ≥1 g/kg, as compared to lower dose < 1 g/kg. Importantly, for the dose group ≥1 g/kg VitC, superior VitC effect [37] as well as reduced toxicity were described [57, 63, 110, 181]. For the dose group < 1 g/kg, several examples that show no added benefit on top of chemotherapeutic agents or even an antagonistic effect were reported [88, 123, 205], highlighting the importance of choosing proper VitC pharmacological doses in vivo, preferably ≥1 g/kg IP, thus reaching sufficient plasma levels to display its anticancer properties [55].

Treatment duration in vitro and frequency in vivo was examined in a similar manner (Fig. 2D). In in vitro studies, cell lines were exposed for long (24-96 h) or short (1–2 h) periods in 74 and 26% of the cases, respectively, generally depending on the type of assay and combination treatment. Although synergism was mostly reported in both cases, short exposures (1–2 h) with a media refresh step are usually preferred to better mimic the physiological conditions in patients [16, 38, 203]. For instance, VitC’s capacity of pH-dependent auto-oxidation and the presence of catalytic metals, such as iron and copper, usually common in cell culture media, can simultaneously increase H_2_O_2_ production and impair reproducibility in vitro [206–208]. In order to further improve reproducibility, a dosing per cell scheme has been shown to correct for H_2_O_2_ toxicity and accumulation in the media [16, 209] (own observations, unpublished data). In conclusion, and in line with its 2 h half-life in patients, in vitro studies should be carried out thoroughly considering ascorbic acid chemistry with recommended experimental conditions such as avoiding catalytic metals in culture media, using a dosing per cell metric scheme and a 2 h treatment with a media refresh step [13, 126, 156].

In vivo, frequency of high dose VitC was reported as daily in the majority of studies (*n* = 21), as well as twice daily (*n* = 6) and twice per week (*n* = 1). All frequency schedules induced enhanced co-treatment efficacy and synergism in a similar manner. Furthermore, in many studies it was unclear whether combination treatments were co-administered or added in a particular sequence. Altogether, what was clear is that successful in vivo studies used ≥1 g/kg IP VitC mostly on a daily basis with a treatment duration ranging from 2 to 8.5 weeks and a median of 3.5 weeks.

It is noteworthy that most of the in vivo studies use ascorbate-synthesizing models, whose human-mimicking features may be questioned. Contrary to humans, mice can synthesize their own VitC, possibly making them suboptimal models for the evaluation of VitC’s anti-cancer effect [55, 210]. As an alternative model, VitC-deficient mice (i.e. Gulo−/− mice) have recently been used to study VitC in cancer as reviewed by Campbell and Dachs [55]. Nevertheless, the different routes of administration and dose ranges from different studies make these two models difficult to compare. Some data suggests that the μM-range VitC basal concentrations in plasma of ascorbate-synthesizing mice (< 100 μM), similar to plasma VitC levels in (healthy) humans with normal dietary VitC uptake, may have only minimal effects on high-dose (mM-range) VitC tumour killing [211–213]. However, considering the low to scurvy-like levels (often < 10 μM) of plasma VitC in many cancer patients 213–215], the use of VitC-deficient mice may be preferred to allow researchers to better fine-tune physiological cancer conditions [56, 213, 216]. An additional remark is that tumour ascorbate levels, instead of plasma levels, might be more relevant to monitor treatment outcome. Direct evidence addressing these issues may help to better evaluate VitC anti-cancer properties and pave the way for promising and robust clinical trials.

### Clinical studies on IVC in combination treatments

Encouraged by the promising results of the pioneering clinical & pre-clinical studies, several phase I and some phase II clinical trials have analysed the use of pharmacologically dosed VitC in combination therapy with conventional cancer treatment agents. A Pubmed database search was performed using search terms “*ascorbate* OR *vitamin C* AND *cancer* AND *clinical trial*”. In total, 34 completed studies were identified (Fig. 3), 16 of which studied medium-to-high dose IVC (Table 2), and 4 focused on IVC monotherapy specifically, as was discussed in earlier sections of this review. In general, these clinical combination studies have focused on a limited number of cancer types, those including high-dose VitC mainly concerning pancreatic cancer, and lower pharmacological doses mainly concerning non-solid tumors (Fig. 3A). An additional search of the clinicaltrials.gov database using search terms *vitamin C* or *ascorbic acid*, and *cancer,* did not reveal any additional trials that were completed with reported results. Many studies were terminated due to a change in standard of care or, more often, because of poor accrual. The large majority of published studies were carried out with only a limited number of patients, and to date, no large-scale, double blind randomized trials that are imperative in determining the clinical efficacy of IVC have been completed. Having said that, 23 clinical trials, including one phase III study, are currently underway, recruiting patients of several cancer types to investigate the effects of adding IVC in a variety of cancer treatment settings. Sixteen of these ongoing studies use medium-to-high dose IVC, and are reported in Table 3.

Most of the clinical studies presented in this section dose-escalated VitC to achieve ≥20 mM plasma ascorbate concentrations. In general, this was achieved when administering 75 g infusions at least 3 times weekly, and was not significantly further increased at 100 g or more [14, 16, 110]. For those studies administering per kg of body weight, amounts ≥1.0 g VitC/kg [151] were needed to achieve plasma levels of at least 20 mM. We focus in detail only on those studies administering ≥1.0 g/kg or ≥ 75 g (high dose) and ≥ 10 g whole body dose (medium dose).

#### Clinical studies combining chemotherapy and radiation therapy

The most studied combination treatment using high-dose IVC is together with chemo- and/or radiotherapy (RT) regimens. Eight such studies were identified, of which half were in conducted in the pancreatic cancer setting (Table 2). As with VitC monotherapy, all studies reported favourable toxicity profiles, with 2 randomized trials specifically observing substantially decreased toxicities compared to control arms without IVC [63, 148], although results of latter study are reported as abstract only without showing data. Both studies administered 75–100 g IVC, Ma et al. [63] 2 times a week for 12 months (of which the first 6 months in conjunction with chemotherapy) and Bruckner et al. [148] 1–2 times per week (with GFLIP every 2 weeks until progression). Compared with RT + temozolomide (TMZ) therapy in a single group study in glioblastoma, the addition of IVC possibly provided a protective effect on hematologic toxicities as judged eg. by incidences of thrombocytopenia reported for similar treatment regimens without IVC in other studies [129]. Importantly, Polireddy et al. [14] found no clinically significant influence on gemcitabine pharmacokinetics, suggesting combination treatment is not detrimental to the mechanism of action of standard of care chemotherapies.

Consistent with positive data obtained from animal and other pre-clinical studies, several of these phase I/II studies reported trends towards increased disease control and objective response rates, although all were underpowered for detection of efficacy. In the randomized trial of Ma et al. [63] in ovarian cancer [63], the median time for disease progression was 8.75 months longer with ascorbate addition to standard chemotherapy (carboplatin and paclitaxel) than in chemotherapy alone. Single group studies showed favourable OS and PFS compared to historical controls [82, 111, 129] and institutional averages [110].

Encouragingly, 2 randomized phase 2 trials are currently ongoing in pancreatic (NCT02905578) [137] and prostate (NCT02516670) [140] cancer patients, directly comparing the added benefit of high-dose IVC to standard chemotherapy. Additionally, 7 single group phase 1 and/or 2 trials studying the combination of high-dose IVC with chemo- and/or chemoradiotherapy are currently underway, among others in lung (NCT02420314 and NCT02905591) [133, 134] and pancreatic (NCT03410030) [139] cancer patients.

#### Clinical studies using VitC in combination with targeted therapy

Three non-randomized clinical studies administered targeted agents on top of chemotherapy and high-dose IVC [151, 153, 155]. Indications of some efficacy were observed in metastatic stage IV pancreatic cancer patients receiving gemcitabine and erlotinib together with IVC [153], with 8/9 patients showing tumour shrinkage after only 8 weeks of treatment. A similar study by Welsh et al. [82], whereby IVC was combined with gemcitabine only, reported similar positive effects, with 6/9 evaluable patients maintaining or improving their performance status. Median overall survival in both studies was 182 days and 13 months, respectively.

Wang et al. [151] combined IVC at 1.5 g/kg once daily for three consecutive days with mFOLFOX6 or FOLFIRI with or without bevacizumab in a 14 day cycle in advanced colorectal and gastric cancer patients (treatment was continued for 12 cycles, disease progression, unmanageable toxic effects, or withdrawal of consent). Besides a favourable safety profile, potential clinical efficacy was observed. Specifically, 14/24 evaluated patients showed PR (objective response rate, ORR, 58.3%) and 9/24 SD (ORR 37.5%), giving a disease control rate of 95.8%. A promising observation was the comparable efficacy in patients with wild-type and with mutant RAS/BRAF tumors. Encouraged by these positive results, this study has since been extended to a randomized phase 3 trial, with an estimated enrolment of 400 mCRC patients (NCT04516681, see Table 3) [131]. To date, this is the only phase 3 trial studying high-dose IVC in anti-cancer treatment.

Ten grade 3 or higher adverse events were reported in the 14 pancreatic cancer patients enrolled in the Monti et al. [153] study, all of which are frequently observed in pancreatic cancer disease progression and/or gemcitabine and erlotinib treatment and thus not likely to be linked to concomitant IVC application. Among the 36 patients enrolled in the Wang et al. study [151], 8 grade 3 or higher adverse events were registered, among which the most common was neutropenia (5 cases), again most likely attributable to the chemotherapy scheme. Likewise, none of the adverse reactions registered in the Kawada et al. [155] study (neutropenia, anemia, and thrombocytopenia) were likely to be directly attributable to IVC treatment.

While all these completed trials studied combinations of chemo- and targeted therapies only, 3 ongoing trials are now investigating the addition of IVC to targeted agents only (eg. in lung cancer patients in randomized trial NCT03799094) [144].

#### Clinical studies using VitC in combination with emerging non-pharmaceutical therapies

Finally, one randomized phase II trial compared a combination of high-dose IVC plus modulated electrohyperthermia (mEHT) with best supportive care (BSC) to BSC alone in advanced stage NSCLC patients. Not only quality of life but also PFS and OS were significantly prolonged in the IVC/mEHT arm (PFS: 3 months vs 1.85 months; OS: 9.4 months vs 5.6 months) [157], suggesting this treatment combination may be a non-toxic way of improving the prognosis of patients with advanced NSCLC. Except for one case of grade 3 diarrhea in the active arm (49 patients), the overall adverse effects of IVC and mEHT were marginal.

## Anti-cancer mechanisms

**Fig. 4:**
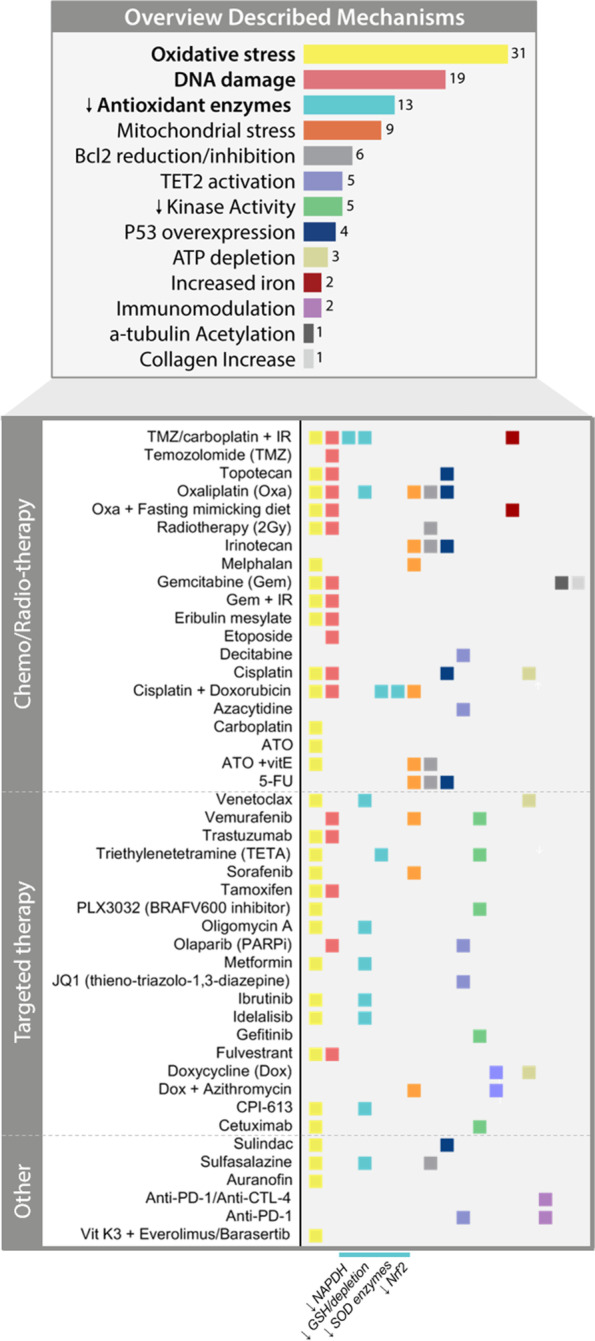
Mechanisms of action described for high-dose VitC in combination with anti-cancer agents in pre-clinical studies. Summary of anti-cancer VitC effects described in vitro and in vivo studies for a total of 45 combinations in the last 5 years (2016–2021). Detailed mechanism of actions per anti-cancer agent are described below. Colour legend corresponds to each mechanism described

The most widely described mechanism by which VitC is cytotoxic to cancer cells in a selective manner is its pro-oxidant facet, which targets redox imbalance. More recent studies have reported additional mechanisms such as epigenome regulation, oxygen-sensing, immunomodulatory functions, epithelial-to-mesenchymal transition and kinase activity regulation [1, 2, 5, 60, 64, 99, 109, 217, 218] (Figs. 4 and 6). Pre-clinical studies studying VitC in combination with other anti-cancer agents have also contributed significantly to the insight into the potential mechanisms of action (MoA) of VitC. By collecting the described MoA from experimental studies dating from 2016 to 2021, we provide an overview of the various cancer modulatory effects that underline VitC as a multi-targeting agent in relation to the treatment (Fig. 4). In total we identified 14 described effects, of which 7 were recurrent (described more than six times). We also generated an up-to-date comprehensive overview of the multi-faceted targeting effects of VitC in the treatment of cancer (Fig. 6).

### Pro-oxidant activity

High concentrations of VitC act as a pro-oxidant, eliciting hydrogen peroxide–dependent cytotoxicity in cancer cells without adversely affecting normal cells [15]. This mechanism is based on VitC redox capacity of metals, such as iron or copper, both generally abundant in tumour cells and involved in important enzyme catalytic activities [219–222]. For instance, reduction of iron from Fe^3+^ to Fe^2+^, known as Fenton reaction, allows the formation of oxygen radicals such as hydrogen peroxide.

In brief, high dose VitC acts as a pro-oxidant in cancer cells; however, in normal cells its anti-oxidant properties are prevalent [2, 54, 63, 181, 223]. One of the causes of cancer cells being more susceptible to high-dose VitC is their increased level of labile iron (Fe^2+^ iron amenable to exchange between reactions), which reacts with H_2_O_2_ to form the damaging hydroxyl radical (OH^•^) [224]. Along with increased iron levels, cancer cells generally have a higher metabolic rate than healthy cells and an abundance of defective mitochondria, leading to endogenously higher oxidative stress levels [16, 225–227]. Moreover, cancer cells generally lack catalase activity, making them extra vulnerable to oxidative stress [2, 228–230]. These anti-cancer effects can be abolished by adding the main detoxifying enzyme catalase to the medium, underscoring a role for H_2_O_2_ [231].

In addition, cancer cells exhibit increased expression of GLUT1. This transporter can also mediate uptake of oxidized VitC (dehydroascorbic acid, DHA) which is reduced back after uptake by the cell, resulting in depletion of intracellular antioxidants such as glutathione (GSH), nicotinamide adenine dinucleotide phosphate (NAPDH) and SOD enzymes, thereby further increasing reactive oxygen species (ROS) levels in cancer cells [32]. Importantly, these anti-cancer effects have been widely reported as synergistic when combining VitC with targeted therapies (Fig. 2).

Therefore, further increasing oxidative stress is an important anti-cancer strategy which also underlines the effectiveness of cytotoxic therapies such as chemo- and radiation therapy.

Many studies have clearly shown that redox functions of VitC are dose-dependent; acting mainly as an anti-oxidant at normal plasma concentrations that range from 30 to 80 μM, and acting as a pro-oxidant in pharmacological concentrations 0.5-20 mM by increasing ROS (i.e. H_2_O_2_ and O_2−_) [1, 232]. High-dose VitC thus leads to ROS formation and thereby targets redox imbalance, which results in DNA, protein and lipid damage of cancer cells [15, 30, 38]. In combination with chemo/radiation therapies, ROS increase, DNA damage, reduction of antioxidant barriers (eg.. SOD2, Nrf2, NAPDH, GSH) and mitochondrial stress were the most reported MoA (Figs. 4 and 6), which may explain the notorious synergistic effect with VitC (Fig. 2).

In addition, four pre-clinical studies reported overexpression of P53 when VitC was combined with chemotherapeutics such as topotecan, oxaliplatin, irinotecan, cisplatin and 5-FU, as well as with anti-inflammatory compound sulindac [88, 101, 118, 120]. Notably, overexpression of P53 gene is known to play a key role in reducing oxidative stress levels by, for instance, mediating enzyme activity of known ROS scavengers glutathione peroxidase (GPX) and aldehyde dehydrogenase (ALDH) [233]. These findings suggest P53 may be involved in VitC-mediated cytotoxicity.

Interestingly, in a study in thyroid cancer, ROS-dependent inhibition of MAPK/ERK and PI3K/AKT pathways has been shown to mediate cancer cytotoxicity in vivo [49]. The synergy between kinase inhibitors and high-dose VitC can be partly explained by increased redox imbalance, considering recent data showing that kinase inhibitors induce synergistic toxicity with low-dose H_2_O_2_ in colorectal cancer cells [234].

Similarly, a remarkable kinase modulator effect was observed in several studies, mostly by reducing phosphorylation levels of ERK, BRAF and AKT [64, 99, 109, 122, 123] (Figs. 2 and 4). This effect might position VitC as a promising alternative to kinase inhibitors in the treatment of cancer.

In addition, the effect of glycolysis inhibitors may also be enhanced by high-dose VitC in a ROS-dependent manner, since both inhibitors increase oxidative stress levels [235]. The efficacy of combining VitC with immunosuppressor auranofin can also be partly ascribed to redox imbalance targeting, since auranofin was shown to induce intracellular accumulation of H_2_O_2_ generated by VitC [236]. Clearly, redox imbalance is a major target involved in the specific anti-cancer activity induced by high-dose VitC.

### Co-factor activity

As mentioned previously, VitC acts as a reducing agent of iron, crucial for Fe-containing protein function. These iron sequestering enzymes are involved in numerous metabolic processes such as the mitochondrial respiratory chain (i.e. cytochrome C, NADH-ubiquinone reductase or complex I), synthesis of collagen (prolyl oxygenase) and oxidative stress regulation (i.e. catalase, peroxidases) [237].

Along with its pro-oxidant function, VitC-mediated cytotoxicity toward cancer cells has also been explained by the 1. regulation of collagen synthesis, 2. hypoxia inducible factor (HIF) proteasomal degradation and 3. TET activity regulation.

#### Collagen synthesis, EMT and invasion

Regulation of collagen synthesis is key for hampering cancer progression. The concept of counteracting decreased collagen synthesis and thereby targeting a potential metastatic vulnerability in cancer by using VitC was first proposed by William McCormick over 60 years ago [238, 239], and subsequently extended by Ewan Cameron [240]. One of the major components of the extracellular matrix are collagen fibrils, which are formed by strong collagen tertiary structures. VitC is known to stabilize these strong cross-links, preventing neoplastic invasion [241, 242]. As mentioned in previous sections, recent pre-clinical studies [14, 33, 39, 43, 58–61] and case report studies [67–72, 243, 244] have shown a significant decrease or depletion of metastasis, and complete tumour regression of advanced or metastatic disease, respectively. Interestingly, Polireddy et al. [14] showed that metastatic reduction in pancreatic cancer was correlated with increased stromal collagen levels in vivo. In their phase I/IIa study, they also found increased collagen levels in a patient who became suitable for tumour resection after 70 doses of IVC (100 g/infusion) and 9 cycles of gemcitabine, compared to untreated, FOLFIRINOX or gemcitabine-treated patients [14].

Another described mechanism by which VitC targets cancer invasion is the reversion of epithelial-to-mesenchymal transition [60, 245]. Zhao et al. [245] reported VitC to inhibit the proliferation, migration and epithelial-mesenchymal-transition of lens epithelial cells through deactivating hypoxia inducible factor. Moreover, Zeng et al. [60] showed a reduction of vimentin and an increase of E-cadherin levels upon high-dose VitC, thereby suppressing EMT and inhibiting cell migration and invasion in breast cancer in vitro and in vivo*.*

In light of collagen synthesis activation, EMT reversion and invasiveness inhibition, high dose VitC could be an effective solution for the prevention and treatment of advanced disease.

#### Oxygen-sensing

Many solid tumours become hypoxic when their growth outruns the emergence of new blood vessels around it. To ensure their survival, tumour cells in turn activate the transcription factor HIF-1 [246, 247].

VitC regulates location and function of HIF hydroxylases, which deactivate HIF-1 by ultimately targeting it to proteasomal degradation and thereby suppressing tumour growth [1, 2, 248–250]. In particular, Fischer and Miles [248] showed that VitC was able to decrease the malignant potential of melanoma by hampering HIF-1α activity, and Kawada et al. [155] showed a downregulation of HIF-1 upon high-dose VitC in human leukemic cells in vitro and in vivo. Jóźwiak et al. [249] also found a negative correlation between HIF-1α mRNA expression and VitC levels in human thyroid neoplastic lesions, suggesting that VitC may also interfere with HIF-1 transcriptional activity. Additional pre-clinical [251, 252] and clinical [253, 254] work by Kuiper and colleagues confirmed this inverse relationship between HIF-1 activity and tumor ascorbate levels. For instance, in their human colorectal cancer study [253], higher levels of tumour VitC were inversely correlated with HIF-1 pathway activation and with a significantly improved disease-free survival. Besides this HIF regulatory function, hypoxia is a common phenomenon in tumour cells and not in normal cells, which increases cancer cell susceptibility to VitC [255].

Given the important role of hypoxia in cancer survival and its well-known implications for treatment resistance, VitC-mediated regulation of HIF activity may provide another facet that is key for improving the treatment of solid tumours.

#### Epigenome regulation

Cancer cells are well known to have aberrant DNA methylation patterns important for survival and tumour progression [256, 257]. Particularly, active DNA demethylation is carried out by the TET enzymes, which are frequently mutated in haematological malignancies. These enzymes are ketoglutarate-, iron- and oxygen-dependent, and belong to the same family as HIF hydroxylases and prolyl hydroxylases crucial for collagen-synthesis as described above.

In the treatment of cancer, high-dose VitC has been shown to induce DNA demethylation by restoring and regulating TET aberrant levels [3]. This anti-cancer VitC role, previously unknown, was widely investigated a couple of years ago in the context of cancer stem cells in leukaemia progression [20, 22]. Sequentially, Vit-C-mediated restoration of TET, also when mutated, enables the re-expression of tumour-suppressor genes in cancer cells [2, 3, 105, 174]. A notable recent study in acute myeloid leukaemia (AML) reported that high-dose VitC activated TET enzymes synergistically with inhibition of mutant isocitrate dehydrogenase 1 (IDH1), resulting in diminished cell growth and increased myeloid differentiation [24].

Vit-C-mediated restoration of TET was also described in four pre-clinical studies combining high dose VitC with chemotherapy [98], targeted therapy [22, 119] and ICI anti-PD-1 [90] (Figs. 4 and 6). Cimmino et al. [22] showed that upon TET2 induced demethylation, high-dose VitC was able to sensitize leukaemia cells to PARP inhibition, mainly due to increased DNA damage.

In addition to TET enzymes, VitC enhances the activity of Jumonji C (JmjC) domain-containing histone demethylases (JHDM) and thereby hinders the aberrant self-renewal of hematopoietic stem cells [3]. Interestingly, these Jumonji histone demethylases are also responsible for epigenetic landscape regulation and for activating cellular responses upon changes in energy metabolism, oxygen and iron levels [219]. In light of the above, VitC can considerably stimulate demethylation in several ways, leading to the re-expression of tumour suppressor genes, and thereby greatly interfering with tumour survival as well as sensitizing to other therapeutic agents.

### Immune modulatory effects

VitC is maintained at high levels in most immune cells and can affect many aspects of the immune response [258]. The contribution of ascorbate as an antioxidant in immune cells is well-established while its cofactor activity for Fe- or Cu-containing oxygenases is emerging as a key factor in the functional effects on both the innate and adaptive immune responses [5, 219]. This activity requires mM concentrations of VitC, thereby emphasizing the need for a high intake to enable adequate immune function, especially in conditions of inflammation and cancer when VitC often becomes deficient. VitC-dependent processes in immune cells include myeloid and T cell differentiation and polarisation, T cell maturation and activation, B cell development, chemotaxis, cytokine production and enhanced NK cell mediated cancer killing [5]. Interestingly, and linked to the previous section, VitC seems to also regulate the epigenetic profile of immune cells such as by TET activity restoration in iTreg cells, which leads to Foxp3 re-expression and drives proper immune cell function [259].

Furthermore, two very recent pre-clinical studies showed that high-dose VitC synergizes with immune checkpoint inhibitors anti-PD-1 and anti-CTL-4 [90, 91] (Figs. 4 and 6). Importantly, Magri et al. [91] observed the largest anti-cancer effect only when administering high-dose VitC to immunocompetent mice and not to immunocompromised mice [91]. This indicates that its anti-tumour activity is not solely dependent on its pro-oxidant effects, but also substantially on some of its immunomodulatory functions.

## Global molecular profiling studies on high-dose IVC in the cancer context

**Fig. 5:**
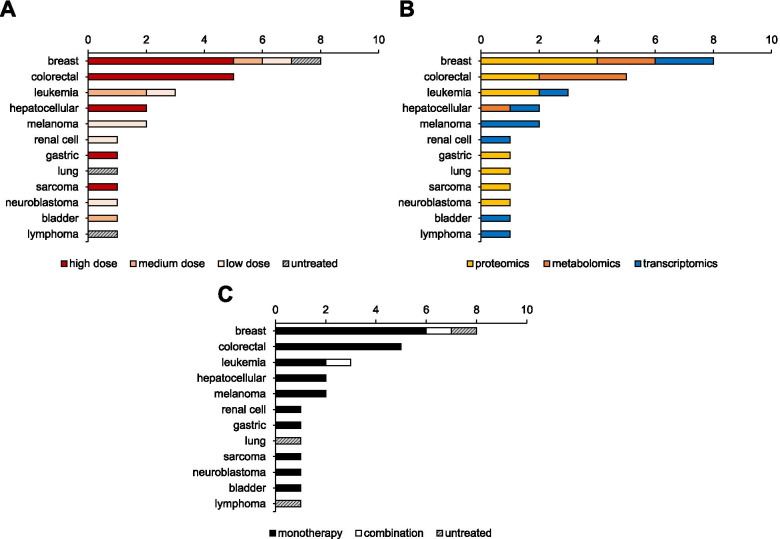
Cancer types studied using global molecular profiling techniques. Annotated are VitC dose group (**A**; high dose ≥1 mM or 1 g/kg, low dose ≤0.1 mM), type of profiling method used (**B**) and treatment type (**C**)

To gain further insights in VitC’s anti-cancer properties on the molecular level, system-wide approaches that capture the complex interplay of various cellular signalling pathways are warranted. Specifically, transcriptomic and especially proteomic studies have the power to capture phenotypic manifestations of genetic alterations. To date, global RNA and protein expression studies on high-dose VitC action are confined to a few cell line studies in specific cancer types. Here, we summarize these studies and their most important findings, considering both studies specifically looking at the global effects of VitC treatment on its own (i.e. without confounding co-treatments), as well as the effects of combining VitC with other (chemo-) therapies (Fig. 5, Table 4).

**Table 4:** Global molecular profiling studies investigating VitC in the cancer context

Cancer type(s)	Model system	Methodology	Treatment(s)	Type of combination therapy	VitC dose^**a**^	Aim	Omics results	Ref.
**Proteomics**								
Colorectal	DiFi (RS and XM Difi) cell lines	SILAC-based MS (LC–ESI–MS-MS)	4 h and 24 h treatments with 1 mM VitCC and/or 50 μg/mL cetuximab	Targeted	high	Hypothesis that VitC in combination with cetuximab could restrain the emergence of secondary resistance to EGFR blockade in CRC RAS/BRAF wild-type models	- Identification of 4147 proteins Switch from glycolysis to oxidative phosphorylation in cetuximab and combo-treated cells at 4 h - downregulation of LDHA/LDHB - upregulation of PDHA1/PDHB and respiratory enzymes Perturbation of iron metabolism in VitC and combo-treated cells at 24 h - downregulation of TFRC - upregulation of FT	[159]
Breast	MDA-MB-231 cell line	Biotin switch approach (enrichment of proteins containing oxidized thiols) followed by LC-MS/MS	30 min treatment with 10 mM ascorbic acid	–	high	Identify early alterations of the redoxome in cellular response to AA that might be linked to AA-induced cell death	- Identification of 2910 cysteine-containing proteins Oxidized targets upon AA treatment: - antioxidant enzymes (eg. PRDX1) - glycolysis and gluconeogenesis pathway (eg. PGK1) - tricarboxylic acid cycle (eg. ACOT7) - DNA, RNA and protein metabolism Cell cycle arrest and translation inhibition associated with AA-induced cytotoxicity. PRDX1 expression levels correlated with AA differential cytotoxicity	[160]
Breast	MCF7 cell line	LC-MS/MS	24 h treatment with 2 mM VitC	–	high	Effect of VitC in itself at different concentration levels on MCF-7 breast cancer cell line	- Identification of 1694 proteins with differential regulation Processes impacted by VitC treatment included - unfolded protein response and inhibition of the cell translation (eIF2α, PKR/PKR pThr-446) - apoptotic process	[161]
Neuroblastoma	SH-SY5Y cell line	SUMO-1 IP followed by ESI-FT ICR MS	30 min treatment with 100 μM ascorbate (or 100 μM hydrogen peroxide)	–	low	Identify redox sensitive proteins of the conjugation machinery for SUMOylation. Oxidative stress (hydrogen peroxide), antioxidant (ascorbate) or control conditions were tested	- Identification of 169 proteins - Great overlap between all treatments - Proteins identified only in the ascorbate sample included DTD2 and MGAT5B - Proteins without predicted SUMOylation site indentified in both ascorabte and hydrogen peroxide treatments included TUBB4A, TUBB1, HNRNPH3, POLG2 and BUB3	[162]
Gastric	AGS cell line	MALDI-TOF MS	24 h treatment with 300 μg/mL (~ 1.7 mM) VitC	–	high	Investigate the molecular mechanism of the inhibitory effect of VitC on AGS cell growth, and protein profiles in AGS cells after exposure to VitC treatment	- 20 differential proteins identified - downregulation eg. of TPM3 and TPM4 - upregulation of PRDX4 and TXND5 - Identified proteins are mainly involved in cell mobility, antioxidant and detoxification, signal transduction and protein metabolism	[163]
Leukemia	NB4 cell line	MALDI–TOF	30 min treatment with 0.5 mM LAA (ascorbic acid)	–	medium	Identification of early protein targets of LAA in leukemia cells	- 9 differential proteins identified - changes in pI as a result of phosphorylation of a TPM isoform) - downregulation eg of of SUPT6H and HSPA8 - upregulation eg. of MATN4 and NONO	[164]
Sarcoma	BALB/C mice implanted with S-180 cancer cells	MALDI TOF-MS/MS	Treatment with 1.5 mg/g body weight ascorbate every three days	–	high	Identify proteins involved in the ascorbic acid-mediated inhibition of tumor progression	- 11 differential proteins identified - upregulastion of RKIP and ANXA5	[165]
Colorectal	BALB/C mice implanted with CT-26 cancer cells	MALDI TOF-MS/MS	Treatment with 1.5 mg/g body weight ascorbate every three days	–	high	Proteome changes of tumor tissue were investigated after intraperitoneal administration of a high concentration of ascorbic acid	- 18 differential proteins identified - upregulation eg. of EIF3I, NPM1 and VIM - regulation of cytoskeleton remodeling	[166]
Breast	MCF7 cell line	LC-MS/MS	18 h treatment with 1 μM DOX (doxorubicin) or DOX + 200 μM of VitC	Chemo	medium	Describe the changes in protein expression and proliferation of the MCF-7 cells induced by the VitC applied with doxorubicin	- Identification of 229 proteins - Downregulation of cytoskeletal (FLNA), ribosomal (eg. RPL27A), transcriptional (eg. HNRNPH1), immune system and antioxidant (HSP90AA1, SOD1) proteins in DOX + VitC-treated cells - Upregulation of GAPDH, GPI and ACTA1	[167]
Leukemia	HL-60 cell line	LC-MS/MS	48 h treatment with 10 μM As2O3 (arsenic trioxide) or As2O3 + 100 μM L-AA (ascorbic acid) + 50 μM α-TOC (α-tocopherol)	Chemo + Dietary suppl.	low	Evaluate the synergistic mechanism of action of vitamins, such as L-ascorbic acid (L-AA) and a-tocopherol (a-TOC) in As2O3 chemotherapy	- Number of identified proteins *n.s.* - Downregulation of cell cycle and translation in cells treated with As2O3, L-AA, and a-TOC compared to As2O3-only - Identification of numerous proteins associated with apoptosis and cell stress in combination treatment	[96]
Breast, Lung	A549 and MDA-MB-231 cell lines	SILAC-based MS (LC-MS/MS)	*untreated* (A549 cell line resistant to 1 mM AUF (auranofin) + 2.5 mM VitC, MDA-MB-231 cell line sensitive)	Anti-inflammatory	*untreated*	Decipher the underlying mechanisms for differential response of lung and breast cancer cell models to redox-modulating molecule auranofin (AUF) and to combinations of AUF and VitC	- Identification of f 4131 proteins common to both cell lines - proteins involved in GSH synthesis and reduction, the pentose phosphate pathway and those belonging to other metabolic pathways (eg PGDH and PTGR1) more abundant in A549 (resistant) cells	[97]
**Transcriptomics**								
Melanoma	A2058 cell line	RNA-seq	48 h treatment with 0.1 mM VitC	–	low	Examined the possible mechanisms that could reveal how VitC suppresses cell migration and anchorage-independent growth of A2058 cells	- 66 genes differentially expressed - alterations predominantly in genes involved in extracellular matrix remodeling. - ARGHAP30, TRIM63 and PTPN7 among 10 most differential genes	[168]
Melanoma	A2058 cell line	RNA-seq	7 days treatment with 100 μM ascorbate	–	low	To elucidate potential mechanism of ascorbate in inducing apoptosis in A2058 cells. Re-analyse data of Gustafson et al., 2015 using updated algorithms	- 344 genes including 20 non-coding RNAs (ncRNA) differentially expressed - expression of CLU gene one of the most downregulated genes	[36]
Breast	MDA-MB-231 cell line	RNA-seq	3 days treatment with 100 μM VitC	–	low	Analysis of transcriptomic changes associated with increased 5hmC generation following exposure to VitC	- 778 differentially expressed genes - TNFSF10, TFRC and PGK1 among 10 most differential genes	[169]
Renal Cell	786-O cell line	RNA-seq	Treatment for 10 passages with 100 μM AsANa (sodium L-ascorbate; VitC) or 100 μM APM (oxidation-resistant VitC derivative)	–	low	Examine ccRCC phenotype changes at the global transcriptome level after treatment of VitC for 10 passages	- 81 differentially expressed genes - most notable genes positively enriched in VitC-treated cells belong to multiple metabolic pathways, such as peroxisome and pentose phosphate pathways - most notable gene sets negatively enriched in VitC-treated cells include DNA replication and mismatch repair genes	[170]
Bladder	T24 cell line	RNA-seq	0.25 mM VitC, *time n.s.*	–	medium	Explore the role of 5hmC in bladder cancer and the therapeutic efficacy of VitC in increasing the 5hmC pattern	- 1172 differentially expressed genes were identified - differential genes mainly associated with focal adhesion, DNA replication, cell cycle, and several cancer-related pathways.	[171]
Hepatocellular	Huh-7 cell line xenograft tumour mouse model	Microarray	3 days treatment of mice with IP injection of 4.0 g/kg or 2.0 g/kg ascorbate	–	high	Assess effects of high-dose ascorbate on hepatoma	- 192 genes/ncRNAs uniquely differentially expressed in HCC tumour tissue obtained from mice treated specifically with high-dose ascorbate (4.0 g/kg/3 days) - deregulated genes were involved in insulin receptor signalling, metabolism and mitochondrial respiration	[172]
Lymphoma	JLPS and JLPR cell lines (sensitive/resistant to ascorbate)	Microarray	*untreated* (JLPR cell line resistant to VitC (incubation of JLPS cells with increasing ascorbate concentrations from 100 μM to 1 mM over 6 month), JLPRS cell line sensitive)	–	*untreated*	Identify possible mechanisms of ascorbate resistance	- Acquired ascorbate resistance associated with downregulation of eg. HMGB1 and MYC and upregulation of eg. ATF5	[173]
Leukemia	HL60 and MOLM13 cell lines	RNA-seq	12 or 72 h treatment with 250 μM L-AA (ascorbic acid)	–	medium	Analyse expression of genes upregulated by Tet2 restoration in cKit+ cells in HL60 and MOLM13 cells treated with L-AA	- 14/50 genes upregulated by Tet2 restoration in mouse cKit+ cells also induced in both human leukemia lines after 12 h of VitC treatment, including genes involved in apoptotic and death receptor signaling (eg. BAX) and NOTCH signaling - Of the top genes downregulated by Tet2-restoration, 34/50 were downregulated in both leukemia lines after 12 h of VitC - Hence, VitC treatment can enhance TET2 function in human leukemia cells in a manner similar to the effects of Tet2 restoration in mouse HSPCs	[174]
Breast	MCF-7 cell line	Microarray	3 days treatment with 100 nM RA (retinoic acid) and/or 1 mM AA (ascorbic acid)	Chemo	high	Elucidate the mechanism by which RA + AA inhibits breast carcinoma proliferation	- 29 genes were up-regulated and 38 genes were down-regulated after RA + AA treatment - up-regulation of antioxidant enzymes (eg. GPX2) and proteins involved in apoptosis (eg. CDK11B), cell cycle regulation (eg. EDN1) and DNA repair (eg. RAD51C) - RA or AA on their own failed to upregulate antioxidant genes	[175]
**Metabolomics**								
Breast, Colorectal	MCF-7, MDA-MB231 and HT29 cell lines	LC-MS	4 h treatment with 3 mM ascorbate	–	high	Gain insight into the cellular effects of high doses of ascorbate	- Metabolic shift, reversal of Warburg effect, disruption of redox homeostasis - Cell death dependent on ascorbate-induced oxidative stress and accumulation of ROS, DNA damage, and depletion of essential intracellular co-factors including NAD+/NADH - disruption of glycolysis, rapid drop in ATP levels - inhibition the TCA cycle and increased oxygen consumption	[176]
Breast, Colorectal	MCF7 and HT29 cell lines	CE-TOF MS	1 h treatment with VitC (0.2 mM, 1 mM or 10 mM)	–	high	Understand anticancer mechanisms of VitC	- Levels of upstream metabolites in the glycolysis pathway and TCA cycle were increased in both cell lines following treatment with VitC - ATP levels decreased concentration-dependently - VitC inhibited energy metabolism through NAD depletion, thereby inducing cancer cell death	[177]
Colorectal	HCT116 and VACO432 cell lines	LC-MS/MS	2 mM VitC for 30 min to 2 h	–	high	Clarify the mechanism by which VitC kills cancer cells while sparing normal cells. Profile metabolic changes following VitC treatment	- Glycolytic intermediates upstream of GAPDH accumulated while those downstream were depleted suggesting that GAPDH was inhibited - Oxidative PPP metabolites increased, indicating that the blockage may shift glycolytic flux into the oxidative PPP - Cysteine, the major limiting precursor for GSH biosynthesis, was also dramatically depleted following VitC treatment - As expected, VitC treatment induced a substantial increase in endogenous ROS in KRAS and BRAF mutant cells	[32]
Hepatocellular	SMMC-7721 cell line	NMR spectroscopy	48 h treatment with 50 μmol/L OXA (oxaliplatin) and/or 1 mmol/L VitC	Chemo	high	Assess the global metabolic changes in HCC cells following VitC treatment	- VitC treatment led to inhibition of energy metabolism via NAD+ depletion and amino acid deprivation - OXA caused significant perturbation in phospholipid biosynthesis and phosphatidylcholine biosynthesis pathways - Glutathione metabolism and pathways related to succinate and choline may play central roles in conferring the combined effect between OXA and VitC	[178]

Twenty-four studies were retrieved from PubMed using search terms (vitamin c OR ascorbate OR ascorbic acid) AND (proteomics OR mass-spectrometry OR metabolomics OR transcriptomics OR RNA-seq OR RNA sequencing OR microarray OR genomics OR DNA sequencing OR WES) AND (cancer). ^a^ *high dose ≥ 1 mM or 1 g/kg, low dose ≤ 0.1 mM*

### Proteomic studies

A number of proteomics studies have been performed to study VitC effects in cancer cell lines employing 2D gel-based analysis and more comprehensive mass spectrometry-based proteomics (Table 4). Here we discuss the latter studies based on nano-liquid-chromatography coupled to mass spectrometry. Very recently, a large-scale proteomic analysis (SILAC-based mass spectrometry) was performed in KRAS/BRAF wild-type CRC cells (DiFi) treated with either VitC (1 mM) or anti-EGFR agent cetuximab, or a combination of both [159]. Both short (4 h) and long-term (24 h) exposure was analyzed. Among the most striking observations was a downregulation of glycolysis in cetuximab and combo-treated cells at early time-points, while proteins related to iron metabolism, such as ferritin and transferrin receptor TFRC, were respectively up and downregulated in VitC and combo-treated cells at later time-points. Based on these results as well as additional metabolic profiling experiments, the authors proposed a model whereby the cetuximab-induced switch from glycolysis to oxidative phosphorylation makes cancer cells more susceptible to the oxidative stress induced by VitC. Subsequent mobilization of iron pools and induction of ROS-mediated stress by VitC could ultimately lead to membrane lipid damage and cell death. A breast cancer study in MDA-MB-231 cells used a biotin switch approach to enrich proteins containing oxidized thiols, followed by LC-MS/MS, to identify very early (30 min) alterations of the redoxome in cellular response to 10 mM ascorbic acid [160]. Besides antioxidant enzymes (such as PRDX1) and glycolysis- and TCA cycle-related proteins (eg. PGK1) showing a significant increase in oxidation upon ascorbic acid treatment, analysis of this redoxome dataset additionally suggested that translation inhibition may be one of the possible mechanisms responsible for oxidative stress-based ascorbic acid cytotoxicity. Using a label-free proteomic approach, another breast cancer study analysed the long-term (24 h) effect of 2 mM VitC on the proteome of MCF-7 cells [161]. Besides proteins directly related to apoptosis, proteins involved in protein processing in the ER were upregulated upon VitC treatment. Specifically, eIF2α and PKR/PKR pThr-446 were suggested to be responsible for the unfolded protein response and inhibition of cell translation during endoplasmic reticulum stress, which may be a direct result of increased oxidative stress. A study focusing on the conjugation machinery for SUMOylation in response to low dose (100 μM) ascorbate performed SUMO-1 IP followed by ESI-FT ICR MS in neuroblastoma cell line SH-SY5Y [162]. This study identified, among others, DTD2 and MGAT5B, two proteins without predicted SUMOylation site, related to translation and glycosylation, respectively, with increased abundance following ascorbate (but not hydrogen peroxide) treatment.

Concerning the effect of combining VitC with other (chemo-) therapies, an LC-MS/MS study in breast cancer cell line MCF7 cell line [167] showed that combining topoisomerase II inhibitor doxorubicin with medium dose (200 μM) VitC lead to a down-regulation of ribosomal, transcriptional and translational, as well and anti-oxidant (eg. SOD1) proteins. Decreased expression of proteins regulating cell cycle and translation was also found when treating HL-60 leukemia cell line with a combination of low dose (100 μM) VitC, ATO and tocopherol (vitamin E) [96]. A SILAC-based mass spectrometry study examined proteomic changes in 2 cell lines (A549 and MDA-MB-231) with different sensitivities to anti-inflammatory redox-modulating molecule auranofin (AUF) in combination with pharmacological doses (2.5 mM) of VitC [97]. Most notably, high expression levels of metabolic proteins with oxidoreductase activity such as TXNRD1, ALDH3A2 and PTGR1 were linked to cellular resistance to AUF/VitC combinations, in line with increased antioxidant mechanisms counteracting the anti-cancer activities of high-dose VitC.

### Transcriptomic studies

Most studies investigating changes in the transcriptome following VitC treatment used doses of less than 1 mM. Three Studies by the same group analysed the effect of 0.1 mM VitC on breast and melanoma cell lines using RNA-sequencing [36, 168, 169]. These analyses revealed, among others, deregulation of apoptotic gene clusterin as well as genes involved in extracellular matrix remodeling in melanoma cell line A2058, as well as increased TNF-related apoptosis-inducing ligand (TRAIL) transcripts in breast cancer cell line MDA-MB-231. Latter study also identified genes related to iron metabolism (TFRC) and glycolysis (PGK1), in line with VitC-induced changes on the protein level observed in the proteomic studies referred to before [159, 160]. Ge and colleagues [170] investigated the effects of long term (10 passages), low-dose (0.1 mM) VitC exposure on renal cell line 786-O, and found that while metabolic processes such as glutathione and pentose-phosphate metabolism were positively enriched, genes related to DNA replication and mismatch repair showed negative enrichment. A similar strong de-regulation of DNA replication-related genes was seen by the same group when treating bladder cancer cell line T24 with medium doses (0.25 mM) of VitC [171]. One notable study focused on the effects of high-dose ascorbate on the transcriptome of Huh-7 cell line xenograft tumour hepatocellular mouse models, as assayed by microarray analysis [172]. Changes in the transcript levels of genes involved in insulin receptor signalling, metabolism and mitochondrial respiration were identified, among which was the upregulation of advanced glycosylation end product-specific receptor (AGER). Possibly related to this are microarray-derived findings on acquired resistance in Lymphoma cell lines by the same group [173]. Here, ascorbate resistant JLPR cells (that were generated by incubation of sensitive JLPS cells with increasing ascorbate concentrations from 0.1 to 1 mM over 6 months) were characterized not only by increased levels of genes such as ferritin, topoisomerase II and glutathione peroxidase 4, but also by the decreased expression of high-mobility group protein box 1 (HMGB1), one of the ligands of AGER. In general, as expected and as seen in several of the proteomic studies, VitC-induced abundancy changes in apoptotic genes are also reported in many of the transcriptomic studies [36, 169, 173–175].

**Fig. 6:**
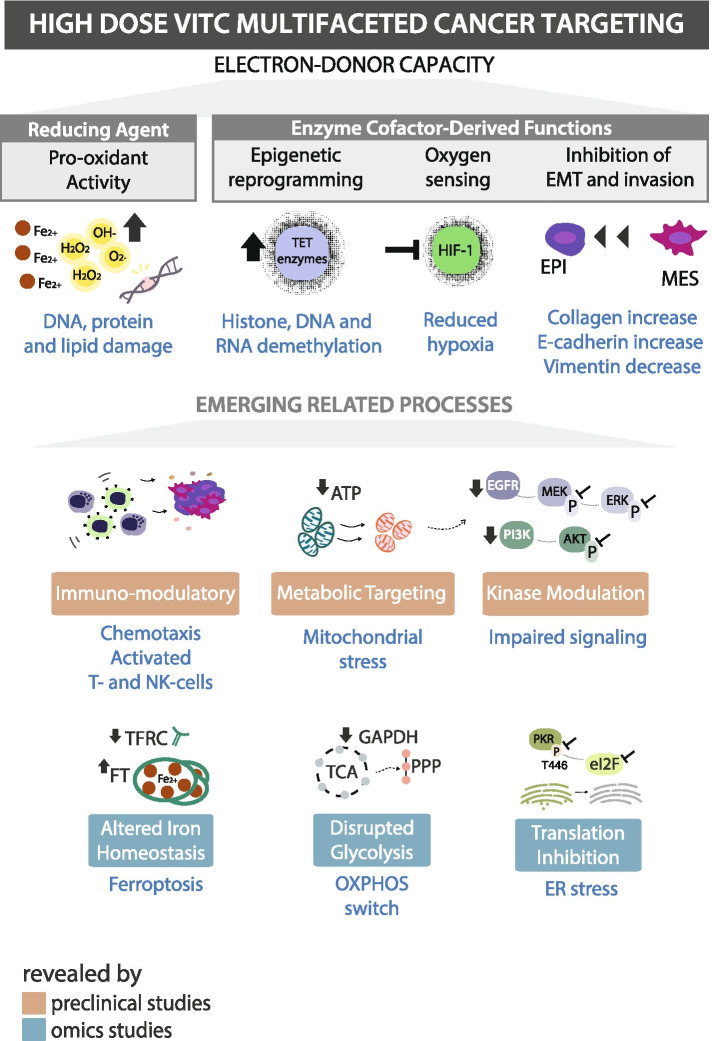
Overview of high-dose VitC multifaceted cancer effects investigated in pre-clinical and omic studies. Schematic representation of the four most known high-dose VitC modulatory effects in cancer cells and the recently concomitant emerging mechanisms

Taken together, both the proteomic and transcriptomic studies identified many known facets of VitC action in cancer cell killing, including apoptotic, redox and metabolic mechanisms, but also revealed less defined roles of ascorbic acid, such as the regulation of cytoskeleton remodeling and the inhibition of translation (proteomics) as well as DNA replication and repair (transcriptomics). The key processes found to be altered in high-dose VitC studies specifically include alteration of iron homeostasis, disruption of glycolysis and inhibition of translation (Fig. 6). In addition, critical proteins involved in these pathways were identified, which may give leads for future (co-) targeting strategies.

### Metabolomic studies

Finally, four studies sought out to globally profile metabolic changes induced by high-dose VitC administration in breast, colorectal and hepatocellular cancer cell line models [32, 176–178]. Although length of treatment and experimental model differed per study, all observed a drop in ATP levels and a depletion of NAD following exposure to high-dose VitC, in line with the inhibition of energy metabolism and multifaceted metabolic rewiring described in numerous pre-clinical studies using alternative approaches. In general, glycolytic metabolites upstream of GAPDH were enriched upon high dose VitC treatment, while those downstream were depleted, in line with an inhibition of GAPDH by VitC, ultimately leading to the disruption of glycolysis and TCA cycle also observed in several proteomics studies (Table 4, Fig. 6).

## Conclusions and outlook

In their 1979 review “Ascorbic Acid and Cancer: A Review” [242], Linus Pauling and colleagues expressed their hopes that “properly designed controlled trials” would soon be conducted to “confirm or refute” their clinical findings, and that if confirmed, “ascorbate will soon become an essential part of all practical cancer treatment and cancer prevention regimens”. Although this vision has not become reality yet, the growing number of well-designed, high impact pre-clinical and early stage clinical studies are contributing to moving the field of high-dose VitC in the cancer care context forward. In addition, with the rise of global profiling strategies such as metabolomics, transcriptomics and large-scale proteomics leading to further delineation of the mechanisms of action of vitamin C, future clinical trials may be designed based on more refined rationales.

Based on molecular characterization of tumor cells, it is becoming increasingly evident that patient subgroups harbouring certain genetic mutations or overexpressing certain proteins may be particularly susceptible to benefiting from VitC mono- and combination therapies. This holds true for tumors baring KRAS mutations for instance, which are generally difficult to treat, being resistant to targeted anti-EGFR therapy amongst others. In this respect, a further boost for the implementation of high-dose VitC in cancer care is expected to arise from an initiative of the Stand Up to Cancer (SU2C) - American Association for Cancer Research charity program, which is raising money for translational cancer research via broad media awareness campaigns. One of the collaborative projects set up as a result of SU2C funding is the “SU2C Colorectal Cancer Dream Team: Targeting Genomic, Metabolic and Immunological Vulnerabilities of Colorectal Cancer”, which has since opened a clinical trial testing the safety and efficacy of high-dose IV VitC as a treatment for KRAS mutant cancers [73]. Importantly, genome sequencing and RNA expression profiling of the tumors collected in this phase II study are planned, in an attempt to further translate pre-clinical mechanistic insights on VitC action to the clinical setting. It has been shown that VitC selectively kills KRAS and BRAF mutant colorectal cancer cells by targeting GAPDH [32], which may also explain why VitC shows to be especially promising in the treatment of pancreatic cancer, where over 90% of the cases harbour KRAS mutations [260] and MM, where RAS family genes also show the most frequent mutations [113]. In addition, tumors baring a TET2 or IDH-1 mutation may be especially sensitive to VitC treatment, and this is also true for cancer types having high concentrations of labile iron, due to low expression of Ferroportin 1 (Fpn1) for instance. Importantly, IDH-1/2 mutations pose an important anti-cancer strategy for hard-to-treat cancer types, these mutations occurring in ~ 70–80% of lower-grade gliomas and the majority of secondary glioblastomas and in up to 20% of patients with AML [261, 262]. Concerning TET2, mutations in this gene are observed in different myeloid malignancies and are related to AML prognosis [263]. In addition, high-dose VitC also has more effect on mismatch repair (MMR)-deficient tumors than on MMR-competent ones, suggesting that the antitumor effect of VitC is enhanced in tumors harbouring increased mutational/neoantigen burdens [91]. Furthermore, sulindac and VitC could be a novel anti-cancer therapeutic strategy for p53 wild-type colon cancers, as this causes apoptosis in a p53-dependent manner [118]. Finally, using high-dose VitC in immune checkpoint therapy may benefit a wide variety of cancer patients, especially those having low PD-1/PDL-1 expression [90].

An absolute necessity in the quest to make high-dose VitC more broadly available to cancer patients is the conduction of randomized phase III clinical trials on large patient groups (typically over 300), with the aim of assessing the effectiveness of VitC (combinations) compared to the current ‘gold standard’ treatment for a given cancer type. Due to their expensive and time-consuming nature, no such trials have been completed for VitC to date. Nevertheless, based on the promising pre-clinical and early-phase clinical trial findings in the colorectal cancer setting [12, 13, 32, 151], a Chinese phase III trial aiming to evaluate the effectiveness of combing high dose IV VitC (1.5 g/kg) with FOLOX +/− bevacizumab versus treatment with FOLFOX +/− bevacizumab alone as first-line therapy in patients with recurrent or advanced colorectal cancer is currently ongoing (ClinicalTrials.gov Identifier: NCT02969681, Table 3, recruiting status unclear). Related to this, another Chinese phase III trial is currently assessing this combination specifically in peritoneal metastatic colorectal cancer patients with high expression of GLUT3 [131] (Table 3).

From a practical point of view, experiences from clinical trials and case reports have made it evident that while adverse events are rare, a few aspects should be considered before administering high doses of IVC. While some side effects, such as a decrease in the levels of potassium (hypokalemia) by VitC may be mitigated by supplementing the formula, certain conditions have to be closely monitored and may be contraindicative for IVC treatment. For example, in patients with renal insufficiency, high dose IVC may lead to kidney stone formation or acute oxalate nephropathy [65, 264], while a red cell glucose-6-phosphate dehydrogenase deficiency (G6PD) has been linked to cases of hemolytic anemia [66, 265] following high dose IVC, sugesting both of these condition should be screened for prior to high dose IVC administration.

Concerning the optimal IVC administration regimen, evidence outlined in this review suggests that 1) anti-cancer effects can only be achieved when VitC is administered intravenously, 2) the dose of IVC has to be sufficiently high in order to generate millimolar concentrations of VitC in the plasma [12, 13]. The recommended effective doses range from 1.5 g/kg [12, 151] to 1.9–2.2 g/kg [13] in the IVC monotherapy studies, while IVC combination therapies indicated 75 g [110, 155] to 87.5 g [16, 129] whole body dose to be sufficient. Furthermore, 3) these doses of IVC should be administered at least twice a week. Almost all clinical trials that present suggestions of efficacy and other favourable clinical outcomes, administered IVC 2–3 times a week, for at least 8 weeks [63, 82, 153, 156, 157].

To conclude, a large body of evidence is accumulating suggesting that VitC, when administered intravenously and in high doses, has potent cancer-selective cytotoxic, cancer-therapy sensitizing and toxicity-reducing properties.

High-dose VitC therefore has the potential to expand the therapeutic range of radio-, chemo- and targeted therapies as well as their efficacy. In addition, a wide variety of cancer patients may benefit from the expanded therapeutic scope of immune checkpoint inhibitors by high-dose VitC. Despite this fact, low accrual remains to hamper further clinical examination, most often because the drug combination in question is no longer standard of care while the study is ongoing. Importantly, this is the case even though the assessment of these combinations may still be highly clinically relevant. Fortunately, future clinical studies combining high-dose VitC with immunotherapy may not face this problem, considering the current high interest in this treatment modality and the need to overcome its current limitations.

Considering how the implementation of high-dose VitC may be a breakthrough in the treatment of cancer patients with poor prognosis and few available treatment options, it is fair to conclude that further clinical examination of this promising and non-toxic cancer treatment modality is not only warranted, but is in fact highly needed.

## Data Availability

Not applicable.

## Abbreviations

3-PO: 3-(3-Pyridinyl)-1-(4-pyridinyl)-2-propen-1-one
5-FU: 5-fluorouracil
AGS: Gastric adenocarcinoma cell line
AML: Acute myeloid leukemia
APC: Antigen-presenting cell
ATO: Arsenic trioxide
AUF: Auranofin
BLAD: Bladder cancer
BR: Breast cancer
BSC: Best supportive care
CLL: Chronic lymphocytic leukemia
CRC: Colorectal cancer
CSCs: Cancer stem cells
Cmax: Peak serum concentration achieved
DHA: Dehydroascorbic acid
DLT: Dose-limiting toxicities
Dox: Doxycycline
d-TPP: TPP derivative dodecyl-TPP
EMT: Epithelial-to-mesenchymal transition
EPI: Epithelial
ESI-FT ICR MS: Electrospray Ionization Fourier Transform Ion Cyclotron Resonance Mass Spectrometry
GB: Glioblastoma
GBM: Glioblastoma multiform
GC: Gastric cancer
Gem: Gemcitabine
HCC: Hepatocellular carcinoma
HNC: Head and neck cancer
ICI: Immune checkpoint inhibitors
IR/RT: Irradiation/radiotherapy
IVC: High-dose intravenous VitC
JQ1: Thieno-triazolo-1,4-diazepine
LC-MS/MS: Liquid chromatography–mass spectrometry
LEU: Leukemia
LIP: Labile iron pool
LUNG: Lung cancer
LYM: Lymphoma
MALDI-TOF MS: Matrix assisted laser desorption ionization-time of flight mass spectrometry
mCRC: Metastatic colorectal cancer
mEHT: Modulated electrohyperthermia
MEL: Melanoma
MES: Mesenchymal
MM: Multiple myeloma
MMR: Mismatch repair
MoA: Mechanisms of action
MTD: Maximum tolerated dose
NB: Neuroblastoma
NSCLC: Non-small-cell lung cancer
OC: Orally administered VitC
ORAL: Oral cancer
ORR: Objective response rate
OS: Overall survival
OVC: Ovarian cancer
Oxa: Oxaplatin
PC: Prostate cancer
PDAC: Pancreatic ductal adenocarcinoma
PFS: Progression-free survival
QoL: Quality of life
RCC: Renal cell carcinoma
RT: Radiation therapy
SAR: Sarcoma
SILAC: STABLE Isotope Labeling by/with Amino acids in Cell culture
TET: Ten eleven translocation enzyme
TETA: Triethylenetetramine
TMZ: Temozolomide
URI: Urinary cancers
VitC: Vitamin C
